# Reliability, and Convergent and Discriminant Validity of Gaming Disorder Scales: A Meta-Analysis

**DOI:** 10.3389/fpsyg.2021.764209

**Published:** 2021-12-07

**Authors:** Seowon Yoon, Yeji Yang, Eunbin Ro, Woo-Young Ahn, Jueun Kim, Suk-Ho Shin, Jeanyung Chey, Kee-Hong Choi

**Affiliations:** ^1^School of Psychology, Korea University, Seoul, South Korea; ^2^Department of Psychology, Seoul National University, Seoul, South Korea; ^3^Department of Psychology, Chungnam National University, Daejeon, South Korea; ^4^Dr. Shin’s Neuropsychiatric Clinic, Seoul, South Korea

**Keywords:** gaming disorder (GD), meta-analysis, convergent validity, discriminant validity, reliability generalization meta-analysis, validity generalization, association

## Abstract

**Background:** An association between gaming disorder (GD) and the symptoms of common mental disorders is unraveled yet. In this preregistered study, we quantitatively synthesized reliability, convergent and discriminant validity of GD scales to examine association between GD and other constructs.

**Methods:** Five representative GD instruments (GAS-7, AICA, IGDT-10, Lemmens IGD-9, and IGDS9-SF) were chosen based on recommendations by the previous systematic review study to conduct correlation meta-analyses and reliability generalization. A systematic literature search was conducted through Pubmed, Proquest, Embase, and Google Scholar to identify studies that reported information on either reliability or correlation with related variables. 2,124 studies were full-text assessed as of October 2020, and 184 were quantitatively synthesized. Conventional Hedges two-level meta-analytic method was utilized.

**Results:** The result of reliability generalization reported a mean coefficient alpha of 0.86 (95% CI = 0.85–0.87) and a mean test-retest estimate of 0.86 (95% CI = 0.81–0.89). Estimated effect sizes of correlation between GD and the variables were as follows: 0.33 with depression (*k* = 45; number of effect sizes), 0.29 with anxiety (*k* = 37), 0.30 with aggression (*k* = 19), –0.22 with quality of life (*k* = 18), 0.29 with loneliness (*k* = 18), 0.56 with internet addiction (*k* = 20), and 0.40 with game playtime (*k* = 53), respectively. The result of moderator analyses, funnel and forest plots, and publication bias analyses were also presented.

**Discussion and Conclusion:** All five GD instruments have good internal consistency and test-retest reliability. Relatively few studies reported the test-retest reliability. The result of correlation meta-analysis revealed that GD scores were only moderately associated with game playtime. Common psychological problems such as depression and anxiety were found to have a slightly smaller association with GD than the gaming behavior. GD scores were strongly correlated with internet addiction. Further studies should adopt a rigorous methodological procedure to unravel the bidirectional relationship between GD and other psychopathologies.

**Limitations:** The current study did not include gray literature. The representativeness of the five tools included in the current study could be questioned. High heterogeneity is another limitation of the study.

**Systematic Review Registration:** [https://www.crd.york.ac.uk/PROSPERO/], identifier [CRD42020219781].

## Introduction

Since games are one of the most popular leisure activities worldwide, they are now available almost everywhere via computers, mobile phones, and tablets. Generally, gamers enjoy gaming as a leisure activity, and the games seem to affect them positively (Jones et al., 2014). Increasing concerns, however, have been raised about excessive gaming behaviors. American Psychiatric Association (2013) has already introduced the provisional diagnostic criteria for internet gaming disorder in Diagnostic and Statistical Manual of Mental Disorders, Fifth Edition (DSM-5). The World Health Organization (WHO) recently adopted gaming disorder (GD) as a diagnosis in the eleventh edition of the International Classification of Diseases (World Health Organization [WHO], 2018). Despite the few discrepancies in the diagnostic criteria for GD in ICD-11 and DSM-5, the common symptoms of GD include continuation of gaming and impaired control over gaming behavior, which result in functional impairments (Jo et al., 2019).

The official listing of GD diagnosis is debatable (Aarseth et al., 2017; Griffiths et al., 2017; Király and Demetrovics, 2017; Kuss et al., 2017; Van Den Brink, 2017; Rumpf et al., 2018; Van Rooij et al., 2018). Several high-quality studies including epidemiological studies (Lemmens et al., 2015; Pontes et al., 2016; Wittek et al., 2016; Han et al., 2018), clinical outcome studies (see King et al., 2017), neuroimaging studies (Fauth-Bühler and Mann, 2017; Han et al., 2017; Liu et al., 2018), and experimental studies (Sariyska et al., 2017; Kräplin et al., 2021) have been published in the recent years, showing improvements with regard to the quality of studies and methodological issues raised by researchers (Petry and O’Brien, 2013; Van Rooij et al., 2018). Most studies, nonetheless, have relied on self-report assessment tools rather than relying on structured clinical interviews, which is partially due to the inconsistency in definition and the different diagnostic criteria (Jeong et al., 2018). Whether the assessment tools are reliable and whether they could validly measure GD are important questions that should be answered.

Another unresolved but important issue is the association between GD and the symptoms of common mental disorders (see Billieux et al., 2017; Van Rooij et al., 2018). Pontes and Griffiths (2019) commented the importance of key risk factors related to comorbidities. Literature has reported mixed results in the association between gaming disorder and psychiatric disorders. Associations between gaming disorder and the common symptoms of mental disorders were found to be considerably weaker than between symptoms of other disorders at least in young age group (Wichstrøm et al., 2018). In contrast, some studies have reported that the underlying mental illness can be a strong predictor of problematic gaming (Kardefelt-Winther, 2014; Billieux et al., 2015), perhaps even a cause (Van Rooij et al., 2018). Authors also have different interpretations for the association. Some authors consider strong association between GD and mental disorders a natural result because clinicians seldomly assess GD without considering comorbidities (Wichstrøm et al., 2019). On the other hand, strong association is also a basis for supporting the idea that GD may be a consequence of other mental disorders (Van Rooij et al., 2018).

In the current study, we focused on construct validity among several aspects of validity since convergent and discriminant validity provide information on the association between GD and other constructs. Reliability and construct validity provide information on what GD assessment tools consistently measure. Poor construct validity of the measure limits the ability of the tools to achieve its intended purpose of measurement because it remains unclear whether the GD instruments represent the construct of the GD or other psychopathological features. If GD instruments have enough construct validity, the association between GD and gaming behavior would be expected to have stronger association compared to the associations between GD and other psychopathological variables.

To our knowledge, no study has systematically examined association between GD scales and symptoms of common psychiatric comorbidities and compared it to the association between GD and gaming behavior. The recent studies on psychological science adopted the reliability generalization and the correlation meta-analytic technique to perform a meta-analysis of a sample of studies with the purpose of estimating the population reliability and population correlation value of the respective studies (Rodriguez and Maeda, 2006; López-Pina et al., 2015; Miller et al., 2018). In the current study, we quantitatively synthesized the bivariate Pearson’s correlation coefficients between GD assessment tools and common psychological problem (e.g., depression, anxiety, aggression) scales, which refers to the statistic of construct validity, to examine the association between GD and psychological variables. We also conducted reliability generalization to examine the consistency of the scales.

Recently, King et al. (2020) reviewed 32 GD assessment tools in their qualitative review paper, recommending five GD instruments with relatively great evidential support. The five tools are 7-item Game Addiction Scale (GAS-7; Lemmens et al., 2009), 9-item Internet Gaming Disorder Scale-Short Form (IGDS9-SF; Pontes and Griffiths, 2015), 10-item Internet Gaming Disorder Test (IGDT-10; Király et al., 2017), Assessment of Internet and Computer Addiction Scale-Gaming (AICA; Müller et al., 2014), and Lemmens Internet Gaming Disorder Scale-9 (Lemmens IGD-9; Lemmens et al., 2015). Among excluded instruments, Young Internet Addiction scale (Young, 1998) is the most frequently utilized scale, and Young Diagnostic Questionnaire (Young, 1998) is the most cited instrument (King et al., 2020). However, they are relatively old scales and are more related to internet addiction rather than GD. In general, YIAT, GAS-7, and IGDS9-SF are frequently used in the field, and IGDT-10 is an instrument that is evenly used in both the West and the East (King et al., 2020). King et al. (2020) recommended the five tools in consideration of the following factors: DSM -5 and ICD-11 coverage, existence of longitudinal studies, adaptation of structured interview, validation of reliability and cut-off score, dimensionality, criterion validity, test refinement and impairment. Divergent validity, however, was not examined by King et al. (2020). Given the importance of the association between GD and other mental disorders, synthesizing and comparing the magnitude of convergent and discriminant validity can significantly contribute to the understanding of GD.

The GD studies often operationalized the convergent validity as there is a bivariate association between a gaming behavior (i.e., hours per week spent gaming) and a score on a GD tool (King et al., 2020). The given association between a score on a GD tool and a gaming behavior represents convergent validity. The associations between the GD tools and other variables can be operationalized as discriminant validity. In a recent article of theirs, Rönkkö and Cho (2020) provided a general definition of discriminant validity. A discriminant validity means that the two measures intended to measure distinct constructs have discriminant validity if the absolute value of the correlation between the measures after correcting for measurement error is low enough for the measures to be regarded as measuring distinct constructs (Rönkkö and Cho, 2020). If the associations between GD and other psychological variables are too strong, the GD tools may reveal the weaknesses in discriminant validity and present the diagnostic needs from the other psychiatric disorders. If the associations are too small, it might not properly reflect the pain and burden of problematic gaming. By quantitatively synthesizing the correlation coefficients to estimate convergent and discriminant validity coefficient, we can quantify and compare the magnitude of each association between GD and other variables.

This study’s objectives are to (1) synthesize the reliability coefficients; (2) examine the convergent and discriminant validity of the GD tools, further investigating the overall association between the GD tools and other psychological/behavioral variables; and (3) investigate how the study characteristics and potential moderator variables affect the reliability and validity estimates, wherein the potential influencing variables include the specific GD instrument used in the study, the type of the sample, study location, and gender ratio of the study participants. Demographic variables such as age, gender, and study location are variables often examined for measurement invariance in this field (see King et al., 2020), and significant moderators of the prevalence rate of GD (see Andreetta et al., 2020; Stevens et al., 2021). Since five scales which cover different domain of diagnostic criteria were included, we did not perform quantitative synthesis on factor structure in order to prevent confusion. Since there is no gold standard for GD diagnosis, and only few studies adopted rigorous clinical interview, we were unable to conduct a meta-analysis for predictive validity of GD assessment tools.

## Methods

### Search Strategy

The current study was conducted based on the PRISMA statement (Moher et al., 2009; Page et al., 2021) and recommendations received for the correlational meta-analyses (Quintana, 2015). PRISMA checklist (Page et al., 2021) is included in Supplementary Material 1. The protocol for the current study has been preregistered on PROSPERO (CRD42020219781). While full electronic search strategy for databases using search terms is a standard procedure for the systematic review, the search strategy in the current study was modified because too many irrelevant and unqualified studies were searched with broad search terms, whereas too many missing studies were searched when narrowing the scope. The first database search for all the published studies with GD assessment tools was executed in PubMed, Proquest, and Embase on August 18, 2020, resulting in 1,343 potentially eligible articles. However, we found too many relevant studies were missing. Great heterogeneity in articles of diagnostic criteria (e.g., DSM-5 and ICD-11 from WHO), type of gaming (e.g., mobile, computer, video-only, online, smartphone gaming), name of the disorder and key-terms (e.g., game addiction, internet gaming, online gaming, video gaming, problematic, overuse, excessive) were factors that made standard search procedure ineffective.

Therefore, we modified our search strategy by selecting a few GD scales to be included in advance. Since King et al. (2020) nicely reviewed 32 GD assessment tools in qualitative way, the five recommended tools with great evidential support were chosen to extract and synthesize the correlation data. The second database search included all the empirical studies that had employed at least one of the 5 GD assessment tools. The search was carried out via two different procedures: (1) A computer-based search of Pubmed, Proquest, and Embase using broad keywords to ensure that all studies adopted one of the five scales are included (e.g., IGDS AND (SF OR short OR 9) not to omit any empirical studies that adopted IGDS9-SF), and (2) a procedural collection of the all Google Scholar citation records for the five tools (as of October 2020). The duplicates of the identified articles were first eliminated by using the Endnote software^1^ version 20 followed by double-checking from the authors. Search strategy of the current study is provided in the Supplementary Material 2.

### Inclusion and Exclusion Criteria

Articles were included if they (a) were peer-reviewed journal articles, (b) used one of the five tools recommended by the current systematic review paper, (c) reported the reliability coefficient or bivariate correlation coefficient via the scales of depression, anxiety, aggression, loneliness, quality of life, internet addiction and game playtime, and (d) written in English. Articles were excluded if they (a) did not include relevant information for GD, (b) non-empirical studies such as meta-analyses and systematic review papers, or (c) did not include the reliability or validity coefficient. Due to difficulties in searching, data extracting, and assessing the study quality, we decided to include the articles which were published with the peer-reviewed process.

### Coding Procedure

All the preselected variables were coded. The coded variables included demographic information of the study, name of the utilized assessment tool, psychometric information, and bivariate Pearson’s correlation coefficient. The potentially eligible articles were systematically coded by three co-authors, namely, SY, YY, and ER. For the longitudinal studies that reported repetitive information using the same sample, we coded the information reported during the first wave. This is because it often contains a larger sample that that during the second or third wave. For multisite cross-sectional studies that included more than one effect size, information for the rest of effect sizes were coded separately. For studies that used various scales to measure only one psychological variable, the effect sizes were integrated into one effect size by calculating the average.

The candidate studies for data synthesis were evenly split between three raters SY, YY, and ER, and then cross-checked by the corresponding author independently. Overall, the level of agreement on the coding was 92.7%, and all the coded information was reached an agreement. A copy of the coding sheet is available in the Supplementary Material 3.

### Selection Process

**FIGURE 1:**
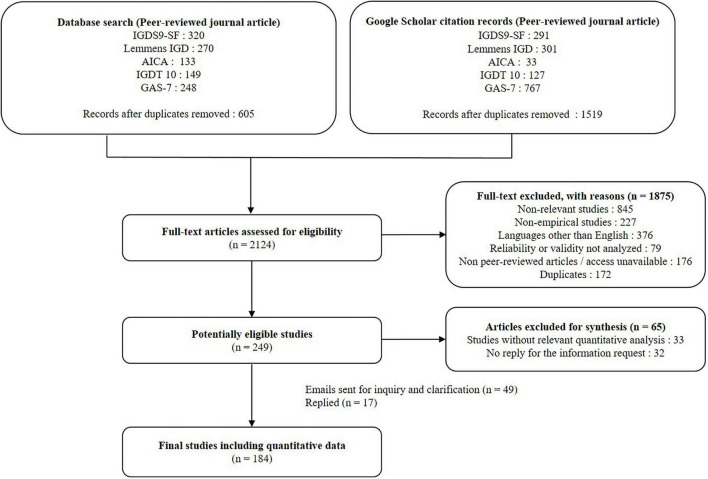
Flow chart of the search process in accordance with PRISMA guidelines.

After the elimination of duplicates using Endnote software, 605 articles were identified via the database keyword search and 1,519 articles were identified via the Google Scholar citation records. Total of 2,124 studies were full-text screened to identify the potentially eligible studies based on the inclusion and exclusion criteria. We found and removed duplicates within and between each database. There were 135 overlapping studies within the Google scholar citation records, and 37 overlapping studies between electronic database search records and Google scholar citation records. As a result, 249 potentially eligible studies were identified. E-mails requesting additional data were sent to the corresponding authors of 49 studies. As of February 2021, 17 authors (34.7%) had responded to the request, and the information provided was finally included in the quantitative synthesis. As a result, 184 of the 249 studies were quantitatively synthesized and 65 were excluded. Among 65 excluded studies, 33 did not include any information on the variables of our interest. The rest 32 studies were excluded due to no reply to the inquiry. Figure 1 presents a flowchart of the database search, screening, and data coding process. The list of the included studies is provided in Supplementary Material 4.

### Meta-Analytic Method and Statistical Analysis for Reliability Generalization

Reliability generalization is a powerful tool to characterize the mean measurement error variance across studies, and also the variabilities in score quality and the study features (Vacha-Haase, 1998). We utilized this technique to estimate the overall level of reliability of the included studies and to find differences in the level of reliability among the five instruments. Separate meta-analyses were conducted for reliability generalization and validity generalization. The current study utilized a meta-analytic technique to quantitatively synthesize the findings of various studies and examine the overall reliability of the GD assessment tools that are frequently used. Cronbach’s alpha coefficients (Cronbach, 1951) were frequently reported, allowing us to synthesize the findings. Information on test-retest reliability, however, was less frequently reported. To conduct reliability generalization of the internal consistency, we extracted the Cronbach’s alpha coefficient for just the total score of the five GD assessment tools drawn from eligible studies. Cronbach’s alpha coefficient offers information on the internal consistency of the test scale (Tavakol and Dennick, 2011). With regard to the calculation of the mean coefficient alpha, Bonett’s (2002) transformation was applied to normalize the distribution and stabilize their variance: Li = Ln (1–αi), where Ln is the natural logarithm. After synthesizing reliability with transformed values, we converted the Bonett-transformed metric back to the original metric of Cronbach’s alpha coefficient to facilitate interpretation. The test-retest reliability coefficients reported from the included studies were descriptively presented in the result. We adopted the same correlation meta-analysis technique for the quantitative synthesis of test-retest reliability coefficients since test-retest reliability is often measured with a correlation coefficient.

### Meta-Analytic Method and Statistical Analysis for Validity Generalization

We coded all bivariate correlation coefficients between GD and psychological variables if the number of effect sizes is sufficient enough to conduct quantitative synthesis (*j* > 10). We considered the correlation between a GD scale score and the game playtime as a convergent validity variable. Depression, anxiety, impulsivity, loneliness, aggression, gambling addiction, internet addiction, alcohol addiction, and quality of life (QOL) were considered potential discriminant validity variables. Among ten variables, gambling addiction (*j* = 5, number of effect sizes), alcohol addiction (*j* = 2), and impulsivity (*j* = 6) were excluded due to the insufficient number of effect sizes for quantitative synthesis. As a result, we performed quantitative synthesis of correlation between GD and seven psychological variables: depression, anxiety, quality of life (QOL), aggression, loneliness, internet addiction, and game playtime.

To estimate the overall mean effect size and correlation coefficient, the current literature has dominantly adopted two approaches (Field and Gillett, 2010; Brannick et al., 2019). These two approaches were proposed by Schmidt and Hunter (1998) and Hedges (Hedges, 1992; Hunter and Schmidt, 2004; Borenstein et al., 2011). However, determining which approach is more appropriate for the correlation coefficient’s meta-analysis has been controversial (Field, 2005; Field and Gillett, 2010). In addition to the two commonly adopted techniques, Brannick et al. (2019) also introduced a novel estimator, providing better coverage and slightly better credibility values than the commonly used approaches. These meta-analytic methods are based on the random-effects model. A random-effects model allows the true effect to differ in each study, whereas a fixed effect model assumes all the studies share a common effect size (Borenstein et al., 2010). As the studies included in this meta-analysis were conducted in different regions and have differ samples, a random effects model was used to derive the effect size and confidence level.

For correcting measurement unreliability, Hunter-Schimidt estimator (Hunter and Schmidt, 2004). Morris estimator (Brannick et al., 2019) apply the individual correction technique to estimate the mean effect size. Hedges method (Borenstein et al., 2011), however, does not adopt the individual correction technique to estimate the effect size. As the current study also aims to conduct reliability generalization to examine the reliability of the GD assessment tools, we utilized the Hedges method. The current study adopted a conventional two-level meta-analytic method instead of a three-level model or robust variance estimation technique to estimate the pooled effect size of the correlation. Although a three-level model and robust variance estimation technique have several advantages over a conventional two-level meta-analytic model (Hedges et al., 2010; Assink and Wibbelink, 2016; Harrer et al., 2021), scarce information on the variance of effect size within individual studies made it difficult to apply a three-level model or robust variance estimation method. We therefore conducted the conventional two-level meta-analysis in the current study.

### Heterogeneity and Moderator Analyses

As the current study synthesized the findings of studies that used five different assessment tools, a high heterogeneity was expected. To examine the heterogeneity of the quantitative synthesis, the current study reported Tau (*T*), Tau-squared (*T*^2^), and *I*^2^ as the measures of heterogeneity between the studies. Tau and Tau-squared are reported in the same metric as the effect size, providing information about the dispersion of true effects on the absolute scale (Borenstein et al., 2017). A guide to interpret the *I*^2^ statistic (Borenstein et al., 2017) is as follows: small heterogeneity (*I*^2^ 25%), moderate heterogeneity (*I*^2^ 50%), and considerable heterogeneity (*I*^2^ = 75%).

Categorical moderator analyses were conducted to identify the potential impacts of reliability and validity generalizations. One study characteristic moderator, (a) the specific GD instrument used in the study (categorized into “IGDS9-SF,” “GAS-7,” “Lemmens IGD-9,” “AICA,” and “IGDT-10”), was considered the potential impact for reliability generalization. Three study characteristics were considered as the potential categorical moderators for validity generalization, namely, (a) the specific GD instrument used in the study, (b) the type of the sample (categorized into “adolescents,” “adults,” and “both”), and (c) the study location (categorized into six continents). Categorical moderator analyses were conducted when each of the subgroups had at least 4 studies. Fu et al. (2011) suggested that each subgroup should have at least four studies for a categorical moderator analysis. Some subgroups with an insufficient number of studies (less than four studies) were excluded from the moderator analysis. To investigate whether the continuous moderator (d) gender ratio affects effect sizes, we performed a meta-regression with the ratio of male participants.

### Statistical Software

The statistical analysis was conducted in R software (version 4.0.3) using metafor (Viechtbauer, 2010), meta (Schwarzer, 2007), and dmetar packages (Harrer et al., 2019). The packages provide various functions to facilitate study synthesis. These include moderator analysis, meta-regression analysis, Egger’s regression test (Egger et al., 1997), sensitivity meta-analysis for publication bias, and various types of meta-analytical plotting.

### Publication Bias

Rothstein et al. (2005) suggested that publication bias, also known as file-drawer problem, could occur since studies without statistically significant results are less likely to be published. The current study examined the risk of publication bias by drawing a funnel plot and conducting Egger’s test (Egger et al., 1997). Egger’s regression test quantifies the funnel plot asymmetry and performs a statistical test. If the *p*-value of Egger’s test is significant, the significant asymmetry in the Funnel plot caused by the publication bias or “small study effects” is indicated (Sterne et al., 2001). Cumulative meta-analysis and sensitivity analysis were additionally conducted when Egger’s test indicated the presence of publication bias.

## Results

### Description of Included Studies

The current study included 184 articles that reported the results from 205 independent samples with 285,752 participants. The estimated mean age of the study samples based on the studies’ reported statistic was 22.12, and 60.7% of the participants were male. Of the studies included up to December 2020, 159 studies (86.4%) have been published since 2016 and 102 studies (55.4%) since 2019. While 94 studies were conducted in Europe, 61 studies were conducted in Asia. Regarding the targeted age group, 63 studies targeted adult samples, 56 studies targeted adolescent samples, and the remaining 65 included both adult and adolescent samples. Of the 184 studies, 49 conducted factor analysis and reported related statistics. While most of the studies (*k* = 42) conducted confirmatory factor analysis, two studies conducted exploratory factor analysis and five studies conducted both. IGDS9-SF was found to be the most frequently utilized tool (*k* = 81, 44.0%). Key characteristics of the included studies are reported in Table 1.

**TABLE 1:** Key characteristics of the included studies for quantitative synthesis.

**Sample size**	** n (%)**
Total	285,752 (100%)
Male	173,570 (60.7%)
Female	112,086 (39.2%)
Unknown	97 (0.0%)

**Characteristics of studies**	** j (%)**

Total	184 (100%)
**Sample target**	
Adults	63 (34.2%)
Adolescents	56 (30.4%)
Both	65 (35.3%)
**Location**	
Europe	94 (51.1%)
Asia	61 (33.2%)
North America	10 (5.4%)
South America	1 (0.5%)
Australia/New Zealand	9 (4.9%)
Africa	1 (0.5%)
Global	8 (4.3%)
**GD tools**	
IGDS9-SF	81 (44.0%)
GAS-7	58 (31.5%)
Lemmens IGD-9	18 (9.8%)
IGDT-10	17 (9.2%)
AICA	10 (5.4%)

*n, number of samples; j, number of studies.*

### Reliability Generalization

#### Result of Reliability Generalization

Cronbach’s alpha coefficient of 193 effect sizes (from 172 studies) were quantitatively synthesized for the respective reliability generalization. The number of studies reporting the Cronbach’s alpha coefficients of GD assessment tools were as follows: 90 effect sizes from the 76 studies for IGDS9-SF, 58 effect sizes from the 53 studies for GAS-7, 20 effect sizes from the 18 studies for Lemmens IGD-9, 16 effect sizes from the 16 studies for IGDT-10 and, 9 effect sizes from the 9 studies for AICA. All the five assessment tools demonstrated an appropriate level of reliability. The estimated average reliability coefficient obtained from Bonett’s transformation was 1.97 (95% CI = 1.90–2.04). Then, to facilitate the interpretation, Bonett’s transformed reliability coefficient was transformed back into Cronbach’s alpha coefficient. The result of RG reported a mean coefficient alpha of 0.86 (95% CI = 0.85–0.87). The result of RG for each of the five GD Assessment Tools is summarized in Table 2. The forest plot of RG is included in the (Supplementary Figure 1).

**TABLE 2:** Result of reliability statistics for the five GD assessment tools.

						**95% CI**	**80% CR**	**Heterogeneity**		
**GD tools**	**j**	**k**	**n**	α_**trf**_	α	**LL, UL**	**LL, UL**	τ	τ^**2**^	**I^2^** **(%)**
Total	172	193	263,979	1.97	0.86	[0.85, 0.87]	[0.64, 0.95]	0.48	0.23	99.3
IGDS9-SF	76	90	65,324	2.20	0.89	[0.88, 0.90]	[0.73, 0.95]	0.45	0.20	98.5
GAS-7	53	58	91,132	1.82	0.84	[0.82, 0.85]	[0.65, 0.92]	0.39	0.15	98.9
Lemmens IGD-9	18	20	16,962	1.64	0.81	[0.76, 0.84]	[0.51, 0.92]	0.46	0.21	98.7
IGDT-10	16	16	54,695	1.70	0.82	[0.77, 0.85]	[0.55, 0.93]	0.45	0.20	99.7
AICA	9	9	35,866	1.92	0.85	[0.80, 0.89]	[0.61, 0.95]	0.47	0.23	99.7

*j, number of studies; k, number of effect size (Cronbach alpha coefficient of GD tools); n, number of samples; α_*trf*_, transformed mean Cronbach’s alpha coefficient; α, back-transformed mean Cronbach’s alpha coefficient; CI, confidence interval; CR, credibility interval; τ, square root of estimated tau^2; τ^2^, estimated amount of total heterogeneity; *I*^2^, total heterogeneity/total variability.*

A total of 8 studies reported test-retest reliability ranging from 0.78 to 0.94. The number of studies reporting the test-retest reliability of the GD assessment tools are as follows: 4 studies for IGDS9-SF (0.78–0.94), 3 studies for GAS-7 (0.80–0.83), 1 study for Lemmens IGD-9 (0.83), and none for IGDT-10 and AICA. The estimated pooled coefficient of test-rest reliability was 0.86 (95% CI = 0.81–0.89).

#### Heterogeneity and Moderator Analysis

The results of the heterogeneity test for reliability were significant for all the included studies (τ = 0.483, τ^2^ = 0.233, *I*^2^ = 99.3%). To assess the effect of the specific GD instrument used in the study on heterogeneity, a categorical moderator analysis on moderator (a) was conducted. Reliability was revealed to be significantly heterogeneous depending on the measure verified via an omnibus test of hypothesis [*QM* (4) = 57.56, *p* < 0.001]. Since IGDS9-SF showed the highest Bonett-transformed coefficient alpha, ANOVA was conducted between the measures. All ANOVA comparisons were conducted to examine whether significant difference exists between the magnitude of each coefficient. The results show that the Bonett-transformed coefficient alpha of IGDS9-SF was significantly higher than the coefficients of GAS-7, Lemmens IGD-9, and IGDT-10 (all *p* < 0.001) but was not higher than the coefficient of AICA (*p* = 0.06). The ANOVA result between AICA and Lemmens IGD-9 was also statistically significant (*p* < 0.05).

#### Publication Bias

Publication bias was assessed using funnel plots and Egger’s regression test. The result of Egger’s regression test did not indicate the presence of publication bias for IGDS9-SF (*z* = 1.37, *p* = 0.17), Lemmens IGD-9 (*z* = –0.76, *p = 0.*45), IGDT-10 (*z* = –0.76, *p* = 0.45), and AICA (*z* = 0.03, *p* = 0.97). Egger’s test of GAS-7, however, indicated the presence of publication bias (*z* = –2.02, *p* = 0.04). Funnel plots are included in the (Supplementary Figure 2).

### Association and Validity Generalization

#### Results of Validity Generalization

A total of 210 effect sizes were extracted and synthesized for validity generalization from the 115 studies analyzed. The number of studies reporting the correlation coefficients between GD assessment tools and psychological or behavioral measurement are as follows: 45 effect sizes from the 44 studies for depression, 37 effect sizes from the 36 studies for anxiety, 19 effect sizes from the 17 studies for aggression, 18 effect sizes from the 17 studies for quality of life and loneliness, 20 effect sizes from the 18 studies for internet addiction, and 53 effect sizes from the 51 studies for game playtime. DASS-21(Depression Anxiety Stress Scales), developed by Antony et al. (1998), is the most frequently utilized psychological scale for depression (*k* = 8) and anxiety (*k* = 8). The Satisfaction with Life Scale (SWLS) for quality of life (*k* = 13), Buss-Perry Aggression Questionnaire (BPAQ) for aggression (*k* = 8), UCLA Loneliness Scale for loneliness (*k* = 16) and Young’s Internet Addiction Test (*k* = 10) for internet addiction were also frequently utilized (Russell et al., 1980; Diener et al., 1985; Buss and Perry, 1992; Young, 1998).

**FIGURE 2:**
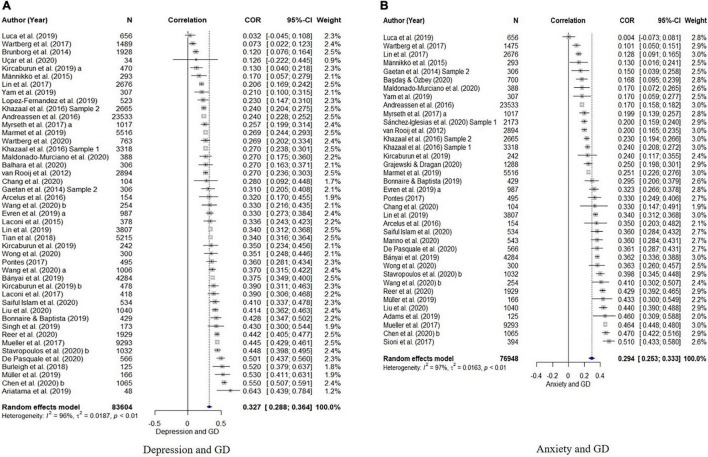
Forest plots of correlations and 95% confidence interval (CI) for random effects meta-analysis model for **(A)** depression, and **(B)** anxiety.

**FIGURE 3:**
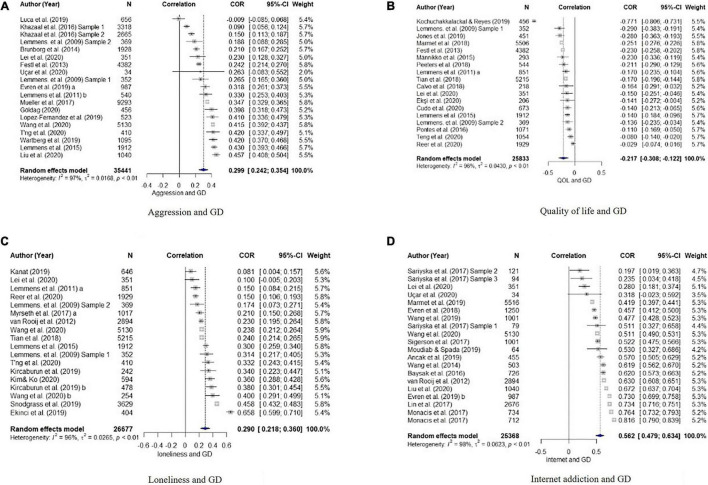
Forest plots of correlations and 95% confidence interval (CI) for random effects meta-analysis model for **(A)** aggression, **(B)** quality of life, **(C)** loneliness, and **(D)** internet addiction.

**FIGURE 4:**
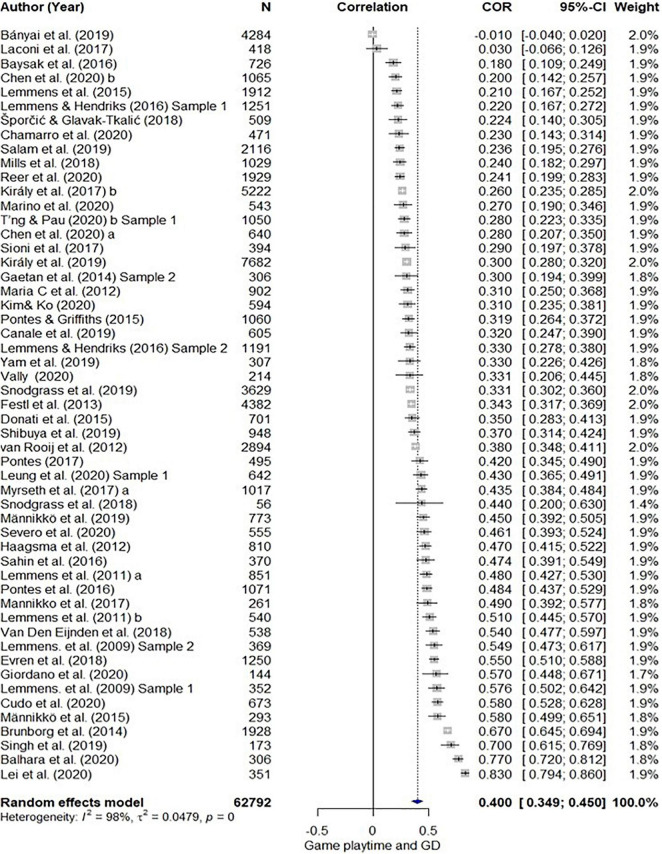
Forest plot of correlations and 95% confidence interval (CI) for random effects meta- analysis model for game playtime.

The results of the quantitative synthesis for the association between GD and other variables are shown in Table 3. The overall estimated mean effect sizes of the psychological variables for GD are as follows: Depression (*r* = 0.33), anxiety (*r* = 0.29), aggression (*r* = 0.30), QOL (*r* = –0.22), and loneliness (*r* = 0.29). The estimated effect sizes of internet addiction and game playtime are *r* = 0.56 and *r* = 0.40. The forest plots displaying the population estimate and the effect sizes of individual studies for each of the variables are presented in Figures 2–4.

**TABLE 3:** Association between GD and psychological/behavioral variables.

					**95% CI**	**Heterogeneity**		
**Psychological variables**	**j**	**k**	**n**	**r_obs_**	**LL, UL**	τ	τ^**2**^	**I^2^** **(%)**
Depression	44	45	83,604	0.33	[0.29, 0.36]	0.14	0.019	95.6
Anxiety	36	37	76,948	0.29	[0.25, 0.33]	0.13	0.016	97.2
Aggression	17	19	35,441	0.30	[0.24, 0.35]	0.13	0.017	96.9
QOL	17	18	25,833	–0.22	[–0.31, –0.12]	0.21	0.043	96.1
Loneliness	17	18	26,677	0.29	[0.22, 0.36]	0.16	0.027	95.8
Internet addiction	18	20	25,368	0.56	[0.48, 0.63]	0.25	0.062	98.2
Game playtime	51	53	62,792	0.40	[0.35, 0.45]	0.22	0.048	97.6

*j, number of studies; k, number of reported effect sizes; n, number of samples; *r*_*obs*_, estimated mean effect sizes (correlation coefficient); *SD*_*r*_, standard deviation for *r*_*obs*_; CI, confidence interval; τ, square root of estimated tau^2; τ^2^, estimated amount of total heterogeneity; *I*^2^, total heterogeneity/total variability.*

#### Heterogeneity and Moderator Analyses

The results of the quantitative synthesis indicated high levels of heterogeneity for all the variables. The heterogeneity estimates are presented in Table 3. Categorical moderator analyses and meta regression analyses using moderators were conducted to identify the potential sources of heterogeneity. Moderator (a), the specific GD instrument used in the study, (categorized into “IGDS9-SF,” “GAS-7,” “Lemmens IGD-9,” “AICA,” and “IGDT-10”), moderator (b), the type of the sample (categorized into “adolescents,” “adults,” and “both”), and (c) the study location (categorized into six continents) were used as the moderators if each of the subgroups had sufficient number of studies (Fu et al., 2011). Moderator (a) was a significant moderator for anxiety and GD (*p* = 0.02), and moderator (c) was a significant moderator for aggression and GD (*p* < 0.01). Moderator (d), gender ratio of the participants of each study, was a significant moderator only for game playtime (*p* = 0.04), indicating that the studies having more male participants reported smaller correlation coefficients between GD and game playtime. The results of the categorical and continuous moderator analysis of validity generalization are presented in the (Supplementary Tables 2, 3).

#### Publication Bias

**FIGURE 5:**
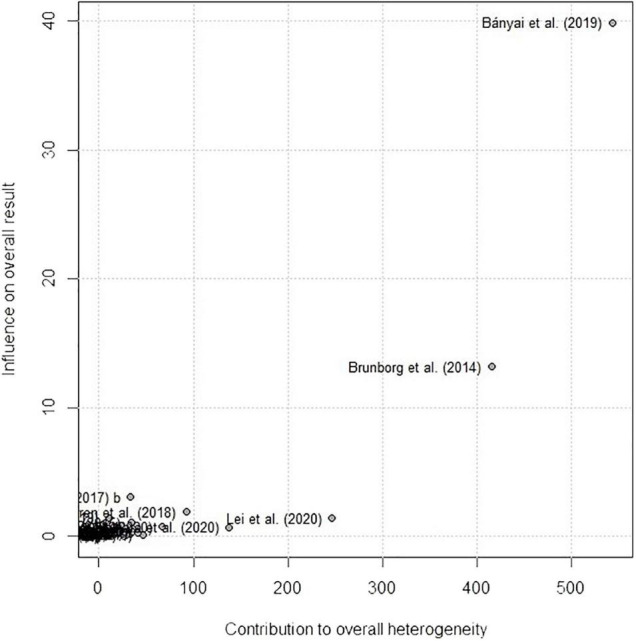
Sensitivity analysis for game playtime and GD.

Publication bias for validity generalization was assessed by using funnel plots and Egger’s regression test. The funnel plots for all the variables have been visualized in Supplementary Figure 3. Since visual inspection can be subjective, Egger’s regression tests for the detection of funnel plot asymmetry were performed (Sterne et al., 2000). The results of the regression tests for game play time were statistically significant (*t* = 3.16, *p* < 0.01), suggesting the presence of evidence for publication bias. Cumulative meta-analysis and sensitivity analysis were further conducted to investigate the publication bias of studies reporting the correlation between GD and game playtime. The results of the cumulative meta-analysis and sensitivity analysis revealed that the studies conducted by, and Brunborg et al. (2014) and Bányai et al. (2019) had influenced the overall effect size estimate as two studies reported exceptionally small and large effect sizes. Omitting study by Brunborg et al. (2014) decreased the overall effect size estimate between GD and game playtime to *r* = 0.39 while omitting study by Bányai et al. (2019) increased the overall effect size estimate to *r* = 0.41. The result of sensitivity analysis for GD and game playtime is provided in Figure 5. The results of Egger’s regression test for the other variables were insignificant (for depression *t* = 0.98, *p* = 0.33; for anxiety *t* = 1.02, *p* = 0.31; for aggression *t* = –0.23, *p* = 0.82; for QOL *t* = –0.37, *p* = 0.72; for loneliness *t* = 0.33, *p* = 0.75; for internet addiction *t* = 0.49, *p* = 0.63).

## Discussion

### Reliability

The current study aimed to provide information on what GD scales measure, and how consistent the measure is. The current study conducted meta-analyses by quantitatively synthesizing the Cronbach’s alpha reliability coefficients and bivariate Pearson’s correlations. The result of the quantitative synthesis of alpha coefficients, reliability generalization, showed an estimated alpha coefficient of 0.86. A high value of alpha coefficient is usually desirable (Cronbach, 1951), but an alpha coefficient above 0.9 may indicate unnecessary redundancy rather than a desirable level of internal consistency (Streiner, 2003). In this regard, the estimated alpha coefficient of 0.86 can be interpreted as an indication of good internal consistency (Gliem and Gliem, 2003). With respect to the moderator analysis, each tool displayed Cronbach’s alpha coefficients ranged from 0.81 to 0.89. The 172 studies in total presented 193 effect sizes of alpha coefficients as the measures of internal consistency. Alpha coefficients of studies with IGDS9-SF were most frequently reported, and the result of ANOVA revealed that IGDS9-SF possesses the highest estimated alpha followed by AICA. The funnel plot and Egger’s test of each GD tool indicated the existence of a potential publication bias for GAS-7 (*z* = –2.02, *p* = 0.04). The funnel plots for the GD tools are provided in the (Supplementary Figure 2).

Given that the current study only included the psychometrically sound tools to synthesize the reliability coefficients, there is a possibility that the reliability estimation of the current study might be positively biased. A categorical moderator analysis with the specific GD instrument used in the study, was performed to examine whether there were differences between each GD tool. The results of the omnibus subgroup test rejected the null hypothesis, indicating that there are differences between the estimated alpha coefficients of each of the tools. ANOVA analyses between every two GD tools were further performed as the omnibus test results increase the type 1 error. The results indicated that IGDS9-SF (α = 0.89) had the highest estimated alpha, followed by AICA (α = 0.85). Lemmens IGD-9 showed the lowest estimated alpha (α = 0.81) among all the tools.

Caution should be taken in interpreting the results of the pooled Cronbach’s alpha coefficient. The high Cronbach’s alpha is not a perfect index of internal consistency as alpha by itself does not assure an excellent degree of internal consistency (Tavakol and Dennick, 2011). An alpha coefficient can be susceptible to the length of the test, undue narrowness (Streiner, 2003), and dimensionality (Green et al., 1977). The test-retest reliability coefficients can provide additional information on overall reliability when they are interpreted together with the internal consistency coefficients. An intraclass correlation coefficient or test-retest interval correlation coefficient can be referred as the stability or reproducibility of the test (Polit, 2014). The estimated average of the eight reliability coefficients was 0.86 (95% CI = 0.81–0.89) which can be interpreted as a good level (Cicchetti, 1994). More studies should examine the test-retest reliability of GD assessment tools as a very small number of studies have reported on retest reliability in comparison to the studies that have reported on internal consistency.

### Validity and Association

The bivariate Pearson’s correlation between the seven variables and GD tools were coded. The estimated effect sizes of the correlation ranged between 0.22 and 0.56 in magnitude. The estimated associations between GD and psychological/behavioral variables were found. The Hedge’s estimator (Borenstein et al., 2011) for the seven variables are as follows: 0.33 for depression, 0.29 for anxiety, 0.30 for aggression, –0.22 for QOL, 0.29 for loneliness, 0.56 for internet addiction, and 0.40 for game playtime.

By synthesizing the effect size of correlation coefficients and examining the convergent and discriminant validity of GD tools, we aimed to scrutinize the association between GD and mental disorders. Unfortunately, the current study offers information only on the association, rather than on causality. The results from the current study do not suggest that the correlation effect sizes are small or large enough to help the society make clear distinction. Since the labeling of the effect size magnitude can be arbitrary (Schober et al., 2018), we suggest an interpretation of the results by comparing each of the effect sizes. For instance, GD tools have a correlation effect size of 0.40 with game playtime and 0.33 with depression, meaning that the depression was found to have a slightly smaller association with GD than the gaming behavior. Anxiety (*r* = 0.29), aggression (*r* = 0.30), and loneliness (*r* = 0.29) showed similar magnitudes of correlation effect sizes. QOL was the only variable negatively associated with GD (*r* = –0.22). Internet addiction showed the highest correlation with GD. The overlapped items between internet addiction and gaming disorder, especially the IGD criteria for DSM-5, might contribute toward a high association between internet addiction and GD.

The results of the moderator analysis show that the specific GD instrument used in the study significantly moderates the correlation between anxiety and GD. IGDS9-SF captures higher associations (*r* = 0.33) between anxiety and GD than GAS-7 (*r* = 0.23). This might be due to the different features of each scales. Study location was found to be a significant moderator for the correlation between aggression and GD. The studies conducted in Asia reported higher association (*r* = 0.38) between aggression and GD than the studies conducted in Europe (*r* = 0.24). This is consistent with the findings of previous studies. Studies reporting the role of aggression in gaming disorders have investigated the mediating role of ethnicity and cultural differences (Kim et al., 2018; Prescott et al., 2018). Anderson et al. (2010) also reported that cultural difference can moderate the association between violence, prosocial behavior, and video gaming. A continuous variable moderator analysis shows that the gender ratio of study participants was a significant moderating continuous variable. The higher the percentage of female participants, the stronger the association between game playtime and GD (*b* = 0.6302 for intercept; *b* = –0.0033 for one percent point increase in the percent of male participants). The males are known to be more vulnerable than females in developing a gaming disorder (Dong et al., 2018; Fam, 2018). The game playtime seems to have a more direct effect on females than on males.

The Egger’s test, cumulative meta-analysis, and sensitivity analysis revealed an asymmetry in the publications reporting the correlations between game playtime and GD. The studies conducted by Brunborg et al. (2014) and Bányai et al. (2019) influenced the overall effect size. Notably, Bányai et al. (2019) reported Pearson’s bivariate correlation between game playtime and GD of *r* = –0.01, which is in essence zero. Since the study by Bányai et al. (2019) included e-sport gamers who spent significantly more time playing games than recreational gamers, the correlation reported by the author significantly differs from that of the other studies. The findings of Bányai et al. (2019) presented the moderating role of gaming motivation in causing GD and psychiatric distress, indicating that gaming behavior itself can have even no association with the GD.

The main findings of the current study show that the magnitudes of the effect sizes of convergent and discriminant validities of GD are not significantly different. Given the association of 0.40 between game playtime and GD, common symptoms (e.g., depression, anxiety) of psychopathology also showed considerable associations with GD. As González-Bueso et al. (2018) commented, we agree to the idea that whether the problematic gaming behaviors are a consequence, or a trigger of other psychopathologies cannot be unraveled yet. Studies have reported that just as problematic gaming increases psychological distress, psychological factors such as low self-esteem and loneliness also bidirectionally affect or predict problematic gaming (Lemmens et al., 2011; Tian et al., 2017; Tras, 2019; Wartberg et al., 2019).

To identify the unraveled relationship between GD and psychopathology, and move beyond these debates, future studies must come to a consensus on the diagnostic criteria of gaming disorder. Delphi method can be helpful in developing the diagnostic criteria of GD and arriving at a consensus (Castro-Calvo et al., 2021). The tools should be improved and unified rather than continuously developed by various researchers. Importantly, the clinician interview must be adopted in this field to verify the positive cases of GD and report comorbid psychopathologies (Pontes and Griffiths, 2019). Of the 184 studies included in the current meta-analysis study, only nine studies included clinical samples and adopted structured clinician interviews in a strict sense (e.g., Müller et al., 2019; Wölfling et al., 2019; Phan et al., 2020). Longitudinal and high-quality clinical trial studies (e.g., Han et al., 2017; Li et al., 2017; Wölfling et al., 2019) are also necessary to rebut the argument that the problematic gaming behavior is a consequence of other psychopathologies. With respect to the other aspects of validity, future studies should actively examine the predictive validity using gold standard tool of the diagnosis.

### Study Limitations

Some limitations should be noted. First, despite our effort to include all the relevant studies, some could not be coded owing to unreported data. To minimize this limitation, we reached out to researchers, and received relevant information from 17 researchers. Second, the current study focuses on the five GD assessment tools recommend by King et al. (2020). Since more than 40 assessment tools have been developed to assess GD, the representativeness of the five tools included in the current study could be questioned. Rather than establishing our own selection criteria, we selected the five GD assessment tools based on a rigorous review article by King et al. (2020). The third limitation might reside in the conventional two-level meta-analysis model and the high level of study heterogeneity found in both reliability and validity generalization. While efforts were made to investigate the potential reason for high heterogeneity, the categorical and continuous moderator analysis only partially adjusted the heterogeneity. We adopted the conventional two-level meta-analysis model instead of three-level model or robust variance estimation method due to scarce report of the variance of the individual effect sizes within each study. We used effect sizes from longitudinal studies (*k* = 17) and several effect sizes reported from the same sample (*k* = 3), and those effect-sizes reported from the same study were not analyzed repeatedly in the current study. If variance of the individual effect sizes within each study are accumulated in a future, a three-level meta-analysis model or robust estimation technique would be recommended to handle the dependent effect sizes and considering within- and between-study heterogeneity. The fourth limitation is that due to insufficient number of studies, we did not perform a meta-analysis for GD and attention deficit hyperactivity disorder, which is a common psychiatric comorbidity in clinical practice (Yen et al., 2017). Five studies reported Pearson’s correlation coefficients ranging from 0.16 to 0.38 between GD and impulsivity. Given the high heterogeneity, we decided that the number of studies on impulsivity was insufficient to carry out a meta-analysis. Fifth, since majority of the included studies in the current study adopted either GAS-7 and IGDS9-SF, the feature of the GAS-7 and IGDS9-SF might affect the effect size estimation. The limitation should be addressed as more studies in this field are conducted.

## Conclusion

Despite its limitations, this is the first and largest systematic review study (with 184 studies and 285,752 study participants) to examine the association between GD and psychological/behavioral variables by synthesizing the reliability, and convergent and discriminant validity information of the five GD assessment tools (e.g., IGDS9-SF, GAS-7, Lemmens IGD-9, AICA, and IGDT-10). In addition to the reliability generalization of the GD assessment tools, a major strength of this study is that we applied meta-analytic techniques to investigate the magnitude of relationships between GD and common symptoms of mental disorders (e.g., depression, anxiety disorders, addictions, impulsivity, and hostility), as indicated in previous studies (Han et al., 2017; Na et al., 2017; Wang et al., 2017; González-Bueso et al., 2018; Liu et al., 2018). We also applied same meta-analytic technique to examine the magnitude of association between GD and the gaming behavior. We believe that this meta-analysis provides current status of GD. Future studies should address debatable issues in reliability and convergent/discriminant validity of the GD assessment tools, and more studies should be conducted to better understand the bidirectional relationship between GD and other psychopathologies.

## Data Availability Statement

The original contributions presented in the study are included in the article/Supplementary Material, further inquiries can be directed to the corresponding author/s.

## Author Contributions

SY, W-YA, JK, S-HS, JC, and K-HC contributed to the conception and design of the study. SY, YY, and ER coded the data and wrote the first draft of the manuscript. SY, YY, ER, and K-HC double-checked the coded data. SY and YY analyzed data. K-HC supervised the overall study process. W-YA, JK, S-HS, JC, and K-HC contributed editing the draft of the manuscript. All authors have read and approved the submitted manuscript.

## Conflict of Interest

The authors declare that the research was conducted in the absence of any commercial or financial relationships that could be construed as a potential conflict of interest.

## Publisher’s Note

All claims expressed in this article are solely those of the authors and do not necessarily represent those of their affiliated organizations, or those of the publisher, the editors and the reviewers. Any product that may be evaluated in this article, or claim that may be made by its manufacturer, is not guaranteed or endorsed by the publisher.

## Footnotes

1
http://endnote.com
