# Supplementary material for: Reliability, and Convergent and Discriminant Validity of Gaming Disorder Scales: A Meta-Analysis
Source: Front Psychol. 2021 Dec 7;12:764209. doi: 10.3389/fpsyg.2021.764209 (PMC8689178; doi:10.3389/fpsyg.2021.764209)
Supplement: Supplementary file 2 [file Table_2.DOCX]

| The number of Google Scholar citation records for GD assessment tools (downloaded all citation records) | | |
| --- | --- | --- |
| IGDS9-SF(~2020.10.17) | | |
|  | Google scholar cited number | 326 |
|  | Non peer-reviewed articles (i.e., Book, poster, thesis, electronic records) | 35 |
|  | Peer-review journal articles | **291** |
|  |  |  |
| Lemmens IGD9(~2020.10.17) | | |
|  | Google scholar cited number | 361 |
|  | Non peer-reviewed articles (i.e., Book, poster, thesis, electronic records) | 60 |
|  | Peer-review journal articles | **301** |
|  |  |  |
| AICAs (~2020.10.17) | | |
|  | Google scholar cited number | 42 |
|  | Non peer-reviewed articles (i.e., Book, poster, thesis, electronic records) | 9 |
|  | Peer-review journal articles | **33** |
|  |  |  |
| IGDT10(~2020.10.17) | |  |
|  | Google scholar cited number | 139 |
|  | Non peer-reviewed articles (i.e., Book, poster, thesis, electronic records) | 12 |
|  | Peer-review journal articles | **127** |
|  |  |  |
| GAS-7 (~2020.10.17) | | |
|  | Google scholar cited number | 988 |
|  | Non peer-reviewed articles (i.e., Book, poster, thesis, electronic records) | 221 |
|  | Peer-review journal articles | **767** |
|  |  |  |
|  | TOTAL | **1519** |

| Database Search keywords for **IGDS9-SF** for peer-reviewed publications | | |
| --- | --- | --- |
| Database 1: Pubmed (2020. 12. 03) | | |
| #1 | IGDS AND (SF OR short OR 9) | 15 |
| #2 | IGDS9 | 24 |
| #3 | (internet gaming disorder scale) AND SF | 30 |
| #4 | (internet gaming disorder scale) AND (short form) | 51 |
| #5 | (nine item) AND IGDS | 9 |
|  | Total | 129 |
| Database 2: Proquest (2020. 12. 03) | | |
| #1 | tiab(internet gaming disorder scale) AND (short OR SF) | 61 |
| #2 | tiab(internet gaming disorder scale) AND (nine item) | 45 |
|  | Total | 106 |
| Database 3: Embase (2020. 12. 03) | | |
| #1 | ('internet gaming disorder scale' OR (('internet'/exp OR internet) AND gaming AND ('disorder'/exp OR disorder) AND ('scale'/exp OR scale))) AND ('sf'/exp OR sf OR short OR nine) | 85 |
|  | Total | 85 |
|  |  |  |
|  | Pubmed + Proquest + Embase | **320** |
|  |  |  |

| Database Search keywords for **Lemmens IGD-9** for peer-reviewed publications | | |
| --- | --- | --- |
| Database 1: Pubmed (2020. 12. 03) | | |
| #1 | igd AND scale AND "9"[tiab] | 30 |
| #2 | "nine item" AND internet AND disorder | 35 |
| #3 | internet gaming AND lemmens | 10 |
|  | Total | 75 |
| Database 2: Proquest (2020. 12. 03) | | |
| #1 | tiab(internet gaming disorder) AND lemmens | 70 |
|  | Total | 70 |
| Database 3: Embase (2020. 12. 03) | | |
| #1 | internet AND gaming AND disorder AND lemmens | 14 |
| #2 | internet AND gaming AND disorder AND scale AND (nine OR 9) NOT form | 111 |
|  | Total | 125 |
|  |  |  |
|  | Pubmed + Proquest + Embase | **270** |
|  |  |  |

| Database Search keywords for **Lemmens IGD-9** for peer-reviewed publications | | |
| --- | --- | --- |
| Database 1: Pubmed (2020. 12. 03) | | |
| #1 | igd AND scale AND "9"[tiab] | 30 |
| #2 | "nine item" AND internet AND disorder | 35 |
| #3 | internet gaming AND lemmens | 10 |
|  | Total | 75 |
| Database 2: Proquest (2020. 12. 03) | | |
| #1 | tiab(internet gaming disorder) AND lemmens | 70 |
|  | Total | 70 |
| Database 3: Embase (2020. 12. 03) | | |
| #1 | internet AND gaming AND disorder AND lemmens | 14 |
| #2 | internet AND gaming AND disorder AND scale AND (nine OR 9) NOT form | 111 |
|  | Total | 125 |
|  |  |  |
|  | Pubmed + Proquest + Embase | **270** |
|  |  |  |

| Database Search keywords for **AICA** for peer-reviewed publications | | |
| --- | --- | --- |
| Database 1: Pubmed (2020. 12. 03) | | |
| #1 | assessment AND internet AND computer AND game AND addiction | 23 |
| #2 | (internet and computer game addiction) AND assessment AND “self report” | 23 |
|  | Total | 46 |
| Database 2: Proquest (2020. 12. 03) | | |
| #1 | (assessment of internet and computer game addiction) AND aica | 18 |
| #2 | (assessment of internet and computer game addiction) AND tiab(igd) | 51 |
|  | Total | 69 |
| Database 3: Embase (2020. 12. 03) | | |
| #1 | aica AND internet AND computer AND game AND addiction | 8 |
| #2 | 'assessment of internet and computer game addiction' | 10 |
|  | Total | 18 |
|  |  |  |
|  | Pubmed + Proquest + Embase | **133** |
|  |  |  |

| Database Search keywords for **IGDT-10** for peer-reviewed publications | | |
| --- | --- | --- |
| Database 1: Pubmed (2020. 12. 03) | | |
| #1 | Internet gaming disorder test AND "10"[tiab] | 42 |
| #2 | IGDT | 13 |
|  | Total | 55 |
| Database 2: Proquest (2020. 12. 03) | | |
| #1 | tiab(internet gaming disorder test) AND ten | 11 |
| #2 | "ten" AND "internet gaming disorder test" | 18 |
| #3 | tiab(IGDT) AND ten | 9 |
|  | Total | 38 |
| Database 3: Embase (2020. 12. 03) | | |
| #1 | 'internet gaming disorder test' | 26 |
| #2 | igdt AND (ten OR 10) | 30 |
|  | Total | 56 |
|  |  |  |
|  | Pubmed + Proquest + Embase | **149** |
|  |  |  |

| Database Search keywords for **GAS-7** for peer-reviewed publications | | |
| --- | --- | --- |
| Database 1: Pubmed (2020. 12. 03) | | |
| #1 | "game addiction scale" | 35 |
| #2 | gas AND game AND (seven or 7) | 9 |
| #3 | 7-Item AND (addiction Scale) | 25 |
| #4 | game AND addiction AND item AND "7"[tiab] | 10 |
|  | Total | **79** |
| Database 2: Proquest (2020. 12. 03) | | |
| #1 | tiab(game addiction scale) and seven | 40 |
| #2 | "7-item" AND "game addiction scale" | 23 |
|  | Total | **63** |
| Database 3: Embase (2020. 12. 03) | | |
| #1 | ('game addiction scale'/exp OR 'game addiction scale' OR (('game'/exp OR game) AND ('addiction'/exp OR addiction) AND ('scale'/exp OR scale))) AND seven | 27 |
| #2 | game AND addiction AND 7 AND item | 79 |
|  | Total | 106 |
|  |  |  |
|  | Pubmed + Proquest + Embase | **248** |
|  |  |  |
