# Supplementary material for: Reliability, and Convergent and Discriminant Validity of Gaming Disorder Scales: A Meta-Analysis
Source: Front Psychol. 2021 Dec 7;12:764209. doi: 10.3389/fpsyg.2021.764209 (PMC8689178; doi:10.3389/fpsyg.2021.764209)
Supplement: Supplementary file 4 [file Table_4.DOCX]

**Supplementary material**

Included studies (APA style)

Adams, B. L., Stavropoulos, V., Burleigh, T. L., Liew, L. W., Beard, C. L., & Griffiths, M. D. (2019). Internet gaming disorder behaviors in emergent adulthood: A pilot study examining the interplay between anxiety and family cohesion. *International Journal of Mental Health and Addiction*, *17*(4), 828-844. doi:10.1089/cpb.2008.0113.

Aggarwal, S., Saluja, S., Gambhir, V., Gupta, S., & Satia, S. P. S. (2020). Predicting likelihood of psychological disorders in PlayerUnknown’s Battlegrounds (PUBG) players from Asian countries using supervised machine learning. *Addictive Behaviors*, *101*, 106132. doi:10.1016/j.addbeh.2019.106132

Ahmed, A., & Sathish, A. (2017). Determinants of behavioral intention use behaviour and addiction towards social network games among Indian college students. *Man in India*, *97*(4), 21-42.

Aleksic, V. (2018). Early adolescents’ digital gameplay preferences, habits and addiction. *Croatian Journal of Education. 20*(2)*,* 463-500. doi:10.15516/cje.v20i2.2583

Andreassen, C. S., Billieux, J., Griffiths, M. D., Kuss, D. J., Demetrovics, Z., Mazzoni, E., & Pallesen, S. (2016). The relationship between addictive use of social media and video games and symptoms of psychiatric disorders: A large-scale cross-sectional study. *Psychology of Addictive Behaviors*, *30*(2), 252-262. doi:10.1037/adb0000160

Andreassen, C. S., Griffiths, M. D., Gjertsen, S. R., Krossbakken, E., Kvam, S., & Pallesen, S. (2013). The relationships between behavioral addictions and the five-factor model of personality. *Journal of Behavioral Addictions*, *2*(2), 90-99. doi:10.1556/jba.2.2013.003

Andreetta, J., Teh MSc, J., Burleigh, T. L., Gomez, R., & Stavropoulos, V. (2020). Associations between comorbid stress and Internet Gaming Disorder symptoms: Are there cultural and gender variations? *Asia‐Pacific Psychiatry*, *12*(2), e12387. doi:10.1111/appy.12387

Arcelus, J., Bouman, W. P., Jones, B. A., Richards, C., Jimenez-Murcia, S., & Griffiths, M. D. (2016). Video gaming and gaming addiction in transgender people: An exploratory study. *Journal of Behavioral Addictions*, *6*(1), 21-29. doi:10.1556/2006.6.2017.002

Ariatama, B., Effendy, E., & Amin, M. M. (2019). Relationship between Internet Gaming Disorder with depressive syndrome and dopamine transporter condition in online games player. *Open Access Macedonian Journal of Medical Sciences*, *7*(16), 2638-2642. doi:10.3889/oamjms.2019.476

Arıcak, O. T., Dinç, M., Yay, M., & Griffiths, M. D. (2018). Adapting the short form of the Internet Gaming Disorder Scale into Turkish: Validity and reliability. *Addicta: The Turkish Journal on Addiction, 6*(1), 15-22. doi:10.15805/addicta.2019.6.1.0027

Baiumy, S., Elella, E. A., Hewedi, D., & Elkholy, H. (2018). Internet gaming disorder scale: Arabic version validation. *Middle East Current Psychiatry*, *25*(1), 13-15. doi:10.1097/01.XME.0000526696.50303

Balhara, Y. P. S., Singh, S., Saini, R., Dahiya, N., Singh, A. B., & Kumar, R. (2021). Should Internet gaming disorder be considered a subtype of generalized problematic internet use? Findings from a study among medical college students. *Perspectives in Psychiatric Care*, *57*(1), 272-278. doi:10.1111/ppc.12558

Bányai, F., Griffiths, M. D., Demetrovics, Z., & Király, O. (2019). The mediating effect of motivations between psychiatric distress and gaming disorder among esport gamers and recreational gamers. *Comprehensive Psychiatry*, *94*, 152117. doi:10.1016/j.comppsych.2019.152117

Başdaş, Ö., & Özbey, H. (2020). Digital game addiction, obesity, and social anxiety among adolescents. *Archives of Psychiatric Nursing*, *34*(2), 17-20. doi:10.1016/j.apnu.2019.12.010

Baysak, E., Kaya, F. D., Dalgar, I., & Candansayar, S. (2016). Online game addiction in a sample from Turkey: Development and validation of the Turkish version of game addiction scale. *Klinik Psikofarmakoloji Bülteni-Bulletin of Clinical Psychopharmacology*, *26*(1), 21-31. doi:10.5455/bcp.20150502073016

Beranuy, M., Machimbarrena, J. M., Vega-Osés, M. A., Carbonell, X., Griffiths, M. D., Pontes, H. M., & González-Cabrera, J. (2020). Spanish validation of the internet gaming disorder scale–short form (IGDS9-SF): Prevalence and relationship with online gambling and quality of life. *International Journal of Environmental Research and Public Health*, *17*(5), 1562. doi:10.3390/ijerph17051562

Bodi, G., Maintenant, C., & Pennequin, V. (2021). The role of maladaptive cognitions in gaming disorder: Differences between online and offline gaming types. *Addictive Behaviors*, *112*, 106595. doi:10.1016/j.addbeh.2020.106595

Bonnaire, C., & Baptista, D. (2019). Internet gaming disorder in male and female young adults: The role of alexithymia, depression, anxiety and gaming type. *Psychiatry Research*, *272*, 521-530. doi:10.1016/j.psychres.2018.12.158

Borzikowsky, C., & Bernhardt, F. (2018). Lost in virtual gaming worlds: Grit and its prognostic value for online game addiction. *The American Journal on Addictions*, *27*(5), 433-438. doi:10.1111/ajad.12762

Brandtner, A., Wegmann, E., & Brand, M. (2020). Desire thinking promotes decisions to game: The mediating role between gaming urges and everyday decision-making in recreational gamers. *Addictive Behaviors Reports*, *12*, 100295. doi:10.1016/j.abrep.2020.100295

Brooks, G. A., & Clark, L. (2019). Associations between loot box use, problematic gaming and gambling, and gambling-related cognitions. *Addictive Behaviors*, *96*, 26-34. doi:10.1016/j.addbeh.2019.04.009

Brunborg, G. S., Hanss, D., Mentzoni, R. A., & Pallesen, S. (2015). Core and peripheral criteria of video game addiction in the game addiction scale for adolescents. *Cyberpsychology, Behavior, and Social Networking*, *18*(5), 280-285. doi:10.1089/cyber.2014.0509

Brunborg, G. S., Mentzoni, R. A., & Frøyland, L. R. (2014). Is video gaming, or video game addiction, associated with depression, academic achievement, heavy episodic drinking, or conduct problems? *Journal of Behavioral Addictions*, *3*(1), 27-32. doi:10.1556/JBA.3.2014.002

Brunborg, G. S., Mentzoni, R. A., Melkevik, O. R., Torsheim, T., Samdal, O., Hetland, J., Andreassen, C. S., & Palleson, S. (2013). Gaming addiction, gaming engagement, and psychological health complaints among Norwegian adolescents. *Media Psychology*, *16*(1), 115-128. doi:10.1080/15213269.2012.756374

Burleigh, T. L., Stavropoulos, V., Liew, L. W., Adams, B. L., & Griffiths, M. D. (2018). Depression, internet gaming disorder, and the moderating effect of the gamer-avatar relationship: An exploratory longitudinal study. *International Journal of Mental Health and Addiction*, *16*(1), 102-124. doi:10.1007/s11469-017-9806-3

Calvo, F., Carbonell, X., Oberst, U., & Fuster, H. (2018). May the passion be with you: The addictive potential of collectible card games, miniatures, and dice of the Star Wars universe. *Journal of Behavioral Addictions*, *7*(3), 727-736. doi:10.1556/2006.7.2018.73

Canale, N., Marino, C., Griffiths, M. D., Scacchi, L., Monaci, M. G., & Vieno, A. (2019). The association between problematic online gaming and perceived stress: The moderating effect of psychological resilience. *Journal of Behavioral Addictions*, *8*(1), 174-180. doi:10.1556/2006.8.2019.01

Carras, M. C., & Kardefelt-Winther, D. (2018). When addiction symptoms and life problems diverge: A latent class analysis of problematic gaming in a representative multinational sample of European adolescents. *European Child & Adolescent Psychiatry*, *27*(4), 513-525. doi:10.1007/s00787-018-1108-1

Chamarro, A., Oberst, U., Cladellas, R., & Fuster, H. (2020). Effect of the frustration of psychological needs on addictive behaviors in mobile videogamers—The mediating role of use expectancies and time spent gaming. *International Journal of Environmental Research and Public Health*, *17*(17), 6429. doi:10.3390/ijerph17176429

Chang, Y.-H., Chang, K.-C., Hou, W.-L., Lin, C.-Y., & Griffiths, M. D. (2021). Internet gaming as a coping method among schizophrenic patients facing psychological distress. *Journal of Behavioral Addictions*, *9*(4), 1022-1031. doi:10.1556/2006.2020.00081

Chen, I.-H., Ahorsu, D. K., Pakpour, A. H., Griffiths, M. D., Lin, C.-Y., & Chen, C.-Y. (2020). Psychometric properties of three simplified Chinese online-related addictive behavior instruments among mainland Chinese primary school students. *Frontiers in Psychiatry*, *11*. doi:10.3389/fpsyt.2020.00875

Chen, I.-H., Strong, C., Lin, Y.-C., Tsai, M.-C., Leung, H., Lin, C.-Y., Pakpour, A. H., & Griffiths, M. D. (2020). Time invariance of three ultra-brief internet-related instruments: Smartphone application-based addiction scale (SABAS), Bergen social media addiction scale (BSMAS), and the nine-item internet gaming disorder scale-short form (IGDS-SF9)(study Part B). *Addictive Behaviors*, *101*, 105960. doi:10.1016/j.addbeh.2019.04.018

Chiu, Y.-C., Pan, Y.-C., & Lin, Y.-H. (2018). Chinese adaptation of the Ten-Item Internet Gaming Disorder Test and prevalence estimate of Internet gaming disorder among adolescents in Taiwan. *Journal of Behavioral Addictions*, *7*(3), 719-726. doi:10.1556/2006.7.2018.92

Collins, E., & Freeman, J. (2013). Do problematic and non-problematic video game players differ in extraversion, trait empathy, social capital and prosocial tendencies? *Computers in Human Behavior*, *29*(5), 1933-1940. doi:10.1016/j.chb.2013.03.002

Collins, E., & Freeman, J. (2014). Video game use and cognitive performance: Does it vary with the presence of problematic video game use? *Cyberpsychology, Behavior, and Social Networking*, *17*(3), 153-159. doi:10.1089/cyber.2012.0629

Cudo, A., Wojtasiński, M., Tużnik, P., Griffiths, M. D., & Zabielska-Mendyk, E. (2020). Problematic Facebook use and problematic video gaming as mediators of relationship between impulsivity and life satisfaction among female and male gamers. *PloS One*, *15*(8), e0237610. doi:10.1371/journal.pone.0237610

de Palo, V., Monacis, L., Sinatra, M., Griffiths, M. D., Pontes, H., Petro, M., & Miceli, S. (2019). Measurement invariance of the nine-item Internet Gaming Disorder Scale (IGDS9-SF) across Albania, USA, UK, and Italy. *International Journal of Mental Health and Addiction*, *17*(4), 935-946. doi:10.1007/s11469-018-9925-5

De Pasquale, C., Dinaro, C., & Sciacca, F. (2018). Relationship of Internet gaming disorder with dissociative experience in Italian university students. *Annals of General Psychiatry*, *17*. doi:10.1186/s12991-018-0198-y

De Pasquale, C., Sciacca, F., Martinelli, V., Chiappedi, M., Dinaro, C., & Hichy, Z. (2020). Relationship of internet gaming disorder with psychopathology and social adaptation in Italian young adults. *International Journal of Environmental Research and Public Health*, *17*(21), 8201. doi:10.3390/ijerph17218201

Donati, M. A., Chiesi, F., Ammannato, G., & Primi, C. (2015). Versatility and addiction in gaming: The number of video-game genres played is associated with pathological gaming in male adolescents. *Cyberpsychology, Behavior, and Social Networking*, *18*(2), 129-132. doi:10.1089/cyber.2014.0342

Dreier, M., Wölfling, K., Duven, E., Giralt, S., Beutel, M. E., & Müller, K. W. (2017). Free-to-play: about addicted whales, at risk dolphins and healthy minnows. monetarization design and internet gaming disorder. *Addictive Behaviors*, *64*, 328-333. doi:10.1016/j.addbeh.2016.03.008

Ekinci, N. E., Ustun, U. D., & Ozer, O. (2016). An investigation of the relationship between digital game addiction, gender and regular sport participation. *Online Submission*, *7*(2), 298-303. doi:10.15503/jecs20162.298.303

Ekinci, N. E., Yalcin, I., & Ayhan, C. (2019). Analysis of loneliness levels and digital game addiction of middle school students according to various variables. *World Journal of Education*, *9*(1), 20-27. doi:10.5430/wje.v9n1p20

Ekinci, N. E., Yalçin, I., Özer, Ö., & Kara, T. (2017). An investigation of the Digital Game Addiction between high school students. *Online Submission*, *14*(4), 4989-4994. doi:10.14687/jhs.v14i4.4936

Ekşi, F., Demirci, İ., & Tanyeri, H. (2020). Problematic technology use and well-being in adolescence: The Personal and relational effects of technology. *Addicta: The Turkish Journal on Addictions, 7*(2), 107-121. doi:10.5152/ADDICTA.2020.19077

Evren, C., Dalbudak, E., Topcu, M., Kutlu, N., Evren, B., & Pontes, H. M. (2018). Psychometric validation of the Turkish nine-item internet gaming disorder scale–short form (IGDS9-SF). *Psychiatry Research*, *265*, 349-354. doi:10.1016/j.psychres.2018.05.002

Evren, C., Evren, B., Dalbudak, E., Topcu, M., & Kutlu, N. (2019). Relationship of internet gaming disorder severity with symptoms of anxiety, depression, alexithymia, and aggression among university students. *Dusunen Adam The Journal of Psychiatry and Neurological Sciences*, *32*(3), 227-235. doi:10.14744/DAJPNS.2019.00032

Evren, C., Evren, B., Dalbudak, E., Topcu, M., & Kutlu, N. (2019). Relationships of Internet addiction and Internet gaming disorder symptom severities with probable attention deficit/hyperactivity disorder, aggression and negative affect among university students. *ADHD Attention Deficit and Hyperactivity Disorders*, *11*(4), 413-421. doi:10.1007/s12402-019-00305-8.

Evren, C., Evren, B., Dalbudak, E., Topcu, M., & Kutlu, N. (2020). Psychometric validation of the Turkish Ten-Item Internet Gaming Disorder Test (IGDT-10). *Dusunen Adam The Journal of Psychiatry and Neurological Sciences*, *33*, 19-28. doi:10.14744/DAJPNS.2019.00057

Evren, C., Pontes, H. M., Dalbudak, E., Evren, B., Topcu, M., & Kutlu, N. (2020). Psychometric validation of the Turkish gaming disorder test: A measure that evaluates disordered gaming according to the World Health Organization framework*. Psychiatry and Clinical Psychopharmacology*, *30*(2), 1-8. doi:10.5455/PCP.20200429072430

Festl, R., Scharkow, M., & Quandt, T. (2013). Problematic computer game use among adolescents, younger and older adults. *Addiction*, *108*(3), 592-599. doi:10.1111/add.12016

Finserås, T. R., Pallesen, S., Mentzoni, R. A., Krossbakken, E., King, D. L., & Molde, H. (2019). Evaluating an internet gaming disorder scale using mokken scaling analysis. *Frontiers in Psychology*, *10*, 911. doi:10.3389/fpsyg.2019.00911

Gaetan, S., Bonnet, A., Bréjard, V., & Cury, F. (2014). French validation of the 7-item Game Addiction Scale for adolescents. *European Review of Applied Psychology*, *64*(4), 161-168. doi:10.1016/j.erap.2014.04.004

Giordano, A. L., Prosek, E. A., Bain, C., Malacara, A., Turner, J., Schunemann, K., & Schmit, M. K. (2020). Withdrawal symptoms among American collegiate internet gamers. *Journal of Mental Health Counseling*, *42*(1), 63-77. doi:10.17744/mehc.42.1.05

Göldag, B. (2020). The relationship between the Digital Game Dependence and Violence Tendency Levels of high school students. *International Education Studies*, *13*(8), 118-129. doi:10.5539/ies.v13n8p118

Gomez, R., Stavropoulos, V., Beard, C., & Pontes, H. M. (2019). Item response theory analysis of the recoded internet gaming disorder scale-short-form (IGDS9-SF). *International Journal of Mental Health and Addiction*, *17*(4), 859-879. doi:10.1007/s11469-018-9890-z

Grajewski, P., & Dragan, M. (2020). Adverse childhood experiences, dissociation, and anxious attachment style as risk factors of gaming disorder. *Addictive Behaviors Reports*, *11*, 100269. doi:10.1016/j.abrep.2020.100269

Haagsma, M. C., King, D. L., Pieterse, M. E., & Peters, O. (2013). Assessing problematic video gaming using the theory of planned behavior: A longitudinal study of Dutch young people. *International Journal of Mental Health and Addiction*, *11*(2), 172-185. doi:10.1007/s11469-012-9407-0

Haagsma, M. C., Pieterse, M. E., & Peters, O. (2012). The prevalence of problematic video gamers in the Netherlands. *Cyberpsychology, Behavior, and Social Networking*, *15*(3), 162-168. doi:10.1089/cyber.2011.0248162

Henchoz, Y., Studer, J., Deline, S., N’Goran, A. A., Baggio, S., & Gmel, G. (2016). Video gaming disorder and sport and exercise in emerging adulthood: A longitudinal study. *Behavioral Medicine*, *42*(2), 105-111. doi:10.1080/08964289.2014.965127

Jones, B. A., Bouman, W. P., Haycraft, E., & Arcelus, J. (2019). The Gender Congruence and Life Satisfaction Scale (GCLS): Development and validation of a scale to measure outcomes from transgender health services. *International Journal of Transgenderism*, *20*(1), 63-80. doi:10.1080/15532739.2018.1453425

Kanat, S. (2019). The relationship between digital game addiction, communication skills and loneliness perception levels of university students. *International Education Studies*, *12*(11), 80-93. doi:10.5539/ies.v12n11p80

Karlsson, J., Broman, N., & Håkansson, A. (2019). Associations between problematic gambling, gaming, and internet use: A cross-sectional population survey. *Journal of Addiction*, *2019*. doi:10.1155/2019/1464858

Khazaal, Y., Breivik, K., Billieux, J., Zullino, D., Thorens, G., Achab, S., Gmel, G., & Chatton, A. (2018). Game addiction scale assessment through a nationally representative sample of young adult men: Item response theory graded–response modeling. *Journal of Medical Internet Research*, *20*(8), e10058. doi:10.2196/10058.

Khazaal, Y., Chatton, A., Rothen, S., Achab, S., Thorens, G., Zullino, D., & Gmel, G. (2016). Psychometric properties of the 7-item game addiction scale among French and German speaking adults. *BMC Psychiatry*, *16*(1), 132. doi:10.1186/s12888-016-0836-3

Kim, B.-N., & Ko, H. (2020). Psychometric properties of the Nine-Item Korean Internet Gaming Disorder Scale: Short form. *Cyberpsychology, Behavior, and Social Networking*, *23*(12), 854-859. doi:10.1089/cyber.2020.0227

Király, O., Bőthe, B., Ramos-Diaz, J., Rahimi-Movaghar, A., Lukavska, K., Hrabec, O., Miovsky, M., Billieux, J., Deleuze, J., & Nuyens, F. (2019). Ten-Item Internet Gaming Disorder Test (IGDT-10): Measurement invariance and cross-cultural validation across seven language-based samples. *Psychology of Addictive Behaviors*, *33*(1), 91-103. doi:10.1037/adb0000433

Király, O., Sleczka, P., Pontes, H. M., Urbán, R., Griffiths, M. D., & Demetrovics, Z. (2017). Validation of the ten-item Internet Gaming Disorder Test (IGDT-10) and evaluation of the nine DSM-5 Internet Gaming Disorder criteria. *Addictive Behaviors*, *64*, 253-260. doi:10.1016/j.addbeh.2015.11.005

Király, O., Tóth, D., Urbán, R., Demetrovics, Z., & Maraz, A. (2017). Intense video gaming is not essentially problematic. *Psychology of Addictive Behaviors*, *31*(7), 807-817. doi:10.1037/adb0000316

Kircaburun, K., Demetrovics, Z., Griffiths, M. D., Király, O., Kun, B., & Tosuntaş, Ş. B. (2020). Trait emotional intelligence and internet gaming disorder among gamers: The mediating role of online gaming motives and moderating role of age groups. *International Journal of Mental Health and Addiction*, *18*(5), 1446-1457. doi:10.1007/s11469-019-00179-x

Kircaburun, K., Griffiths, M. D., & Billieux, J. (2019). Psychosocial factors mediating the relationship between childhood emotional trauma and internet gaming disorder: A pilot study. *European Journal of Psychotraumatology*, *10*(1), 1565031. doi:10.1080/20008198.2018.1565031

Kircaburun, K., Griffiths, M. D., & Billieux, J. (2019). Trait emotional intelligence and problematic online behaviors among adolescents: The mediating role of mindfulness, rumination, and depression. *Personality and Individual Differences*, *139*, 208-213. doi:10.1016/j.paid.2018.11.024

Kochuchakkalackal, G. K., & Reyes, M. E. S. (2019). Compulsive internet gaming and its relationship with poor psychological well-being among selected adolescents. *Journal of Technology in Behavioral Science*, *4*(4), 303-308. doi:10.1007/s41347-019-00098-7

Koning, I. M., Peeters, M., Finkenauer, C., & Van Den Eijnden, R. J. (2018). Bidirectional effects of Internet-specific parenting practices and compulsive social media and Internet game use. *Journal of Behavioral Addictions*, *7*(3), 624-632. doi:10.1556/2006.7.2018.68

Krossbakken, E., Pallesen, S., Mentzoni, R. A., King, D. L., Molde, H., Finserås, T. R., & Torsheim, T. (2018). A cross-lagged study of developmental trajectories of video game engagement, addiction, and mental health. *Frontiers in Psychology*, *9*, 2239. doi:10.3389/fpsyg.2018.02239

Kuriala, G. K., & Reyes, M. E. S. (2020). Efficacy of the Acceptance and Cognitive Restructuring Intervention Program (ACRIP) on the Internet Gaming Disorder symptoms of selected Asian adolescents. *Journal of Technology in Behavioral Science*, *5*, 238-244. doi:10.1007/s41347-020-00132-z

Kuss, D. J., Shorter, G. W., van Rooij, A. J., Griffiths, M. D., & Schoenmakers, T. M. (2014). Assessing internet addiction using the parsimonious internet addiction components model—a preliminary study. *International Journal of Mental Health and Addiction*, *12*(3), 351-366. doi:10.1007/s11469-013-9459-9

Laconi, S., Pirès, S., & Chabrol, H. (2017). Internet gaming disorder, motives, game genres and psychopathology. *Computers in Human Behavior*, *75*, 652-659. doi:10.1016/j.chb.2017.06.012

Laconi, S., Tricard, N., & Chabrol, H. (2015). Differences between specific and generalized problematic Internet uses according to gender, age, time spent online and psychopathological symptoms. *Computers in Human Behavior*, *48*, 236-244. doi:10.1016/j.chb.2015.02.006

Lei, W., Liu, K., Zeng, Z., Liang, X., Huang, C., Gong, K., He, W., Xiang, B., Zhang, J., & Zheng, X. (2020). The psychometric properties of the Chinese version internet gaming disorder scale. *Addictive Behaviors*, *106*, 106392. doi:10.1016/j.addbeh.2020.106392

Lemmens, J. S., & Hendriks, S. J. (2016). Addictive online games: Examining the relationship between game genres and Internet Gaming Disorder. *Cyberpsychology, Behavior, and Social Networking*, *19*(4), 270-276. doi:10.1089/cyber.2015.0415

Lemmens, J. S., Valkenburg, P. M., & Gentile, D. A. (2015). The Internet gaming disorder scale. *Psychological Assessment*, *27*(2), 567-582. doi:10.1037/pas0000062

Lemmens, J. S., Valkenburg, P. M., & Peter, J. (2009). Development and validation of a game addiction scale for adolescents. *Media Psychology*, *12*(1), 77-95. doi:10.1080/15213260802669458

Lemmens, J. S., Valkenburg, P. M., & Peter, J. (2011). The effects of pathological gaming on aggressive behavior. *Journal of Youth and Adolescence*, *40*(1), 38-47. doi:10.1007/s10964-010-9558-x

Lemmens, J. S., Valkenburg, P. M., & Peter, J. (2011). Psychosocial causes and consequences of pathological gaming. *Computers in Human Behavior*, *27*(1), 144-152. doi:10.1016/j.chb.2010.07.015

Leung, H., Pakpour, A. H., Strong, C., Lin, Y.-C., Tsai, M.-C., Griffiths, M. D., Lin, C.-Y., & Chen, I.-H. (2020). Measurement invariance across young adults from Hong Kong and Taiwan among three internet-related addiction scales: Bergen social media addiction scale (BSMAS), smartphone application-based addiction scale (SABAS), and internet gaming disorder scale-short form (IGDS-SF9)(study Part A). *Addictive Behaviors*, *101*, 105969. doi:10.1016/j.addbeh.2019.04.027

Liew, L. W., Stavropoulos, V., Adams, B. L., Burleigh, T. L., & Griffiths, M. D. (2018). Internet gaming disorder: The interplay between physical activity and user–avatar relationship. *Behaviour & Information Technology*, *37*(6), 558-574. doi:10.1080/0144929X.2018.1464599

Lin, C.-Y., Broström, A., Nilsen, P., Griffiths, M. D., & Pakpour, A. H. (2017). Psychometric validation of the Persian Bergen Social Media Addiction Scale using classic test theory and Rasch models. *Journal of Behavioral Addictions*, *6*(4), 620-629. doi:10.1556/2006.6.2017.071

Lin, C.-Y., Imani, V., Broström, A., Årestedt, K., Pakpour, A. H., & Griffiths, M. D. (2019). Evaluating the psychometric properties of the 7-item Persian Game Addiction Scale for Iranian adolescents. *Frontiers in Psychology*, *10*, 149. doi:10.3389/fpsyg.2019.00149.

Lin, C.-Y., Imani, V., Broström, A., Nilsen, P., Fung, X. C., Griffiths, M. D., & Pakpour, A. H. (2019). Smartphone application-based addiction among Iranian adolescents: A psychometric study. *International Journal of Mental Health and Addiction*, *17*(4), 765-780. doi:10.1007/s11469-018-0026-2

Liu, Y., Wang, Q., Jou, M., Wang, B., An, Y., & Li, Z. (2020). Psychometric properties and measurement invariance of the 7-item game addiction scale (GAS) among Chinese college students. *BMC Psychiatry*, *20*, 484. doi:10.1186/s12888-020-02830-7

Lopez-Fernandez, O., Williams, A. J., & Kuss, D. J. (2019). Measuring female gaming: gamer profile, predictors, prevalence, and characteristics from psychological and gender perspectives. *Frontiers in Psychology*, *10*, 898. doi:10.3389/fpsyg.2019.00898

Luca, C., Guicciardi, M., Maria, S., Lucia, M., Alessandra, S., & Silvia, C. (2019). The use of digital technologies, impulsivity and psychopathological symptoms in adolescence. *Behavioral Sciences*, *9*(8), 82. doi:10.3390/bs9080082

Macey, J., & Hamari, J. (2018). Investigating relationships between video gaming, spectating esports, and gambling. *Computers in Human Behavior*, *80*, 344-353. doi:10.1016/j.chb.2017.11.027

Maftei, A., & Enea, V. (2020). Symptoms of Internet Gaming Disorder and parenting styles in Romanian adolescents. *Psihologija*, *53*(3), 307-318. doi:10.2298/PSI190808008M

Maldonado-Murciano, L., Pontes, H. M., Griffiths, M. D., Barrios, M., Gómez-Benito, J., & Guilera, G. (2020). The Spanish Version of the Internet Gaming Disorder Scale-Short Form (IGDS9-SF): Further Examination Using Item Response Theory. *International Journal of Environmental Research and Public Health*, *17*(19), 7111. doi:10.3390/ijerph17197111

Männikkö, N., Billieux, J., & Kääriäinen, M. (2015). Problematic digital gaming behavior and its relation to the psychological, social and physical health of Finnish adolescents and young adults. *Journal of Behavioral Addictions*, *4*(4), 281-288. doi:10.1556/2006.4.2015.040

Männikkö, N., Billieux, J., Nordström, T., Koivisto, K., & Kääriäinen, M. (2017). Problematic gaming behaviour in Finnish adolescents and young adults: relation to game genres, gaming motives and self-awareness of problematic use. *International Journal of Mental Health and Addiction*, *15*(2), 324-338. doi:10.1007/s11469-016-9726-7

Männikkö, N., Ruotsalainen, H., Tolvanen, A., & Kääriäinen, M. (2019). Psychometric properties of the Internet Gaming Disorder Test (IGDT‐10) and problematic gaming behavior among Finnish vocational school students. *Scandinavian Journal of Psychology*, *60*(3), 252-260. doi:10.1111/sjop.12533

Marino, C., Canale, N., Vieno, A., Caselli, G., Scacchi, L., & Spada, M. M. (2020). Social anxiety and Internet gaming disorder: The role of motives and metacognitions. *Journal of Behavioral Addictions*, *9*(3), 617-628. doi:10.1556/2006.2020.00044

Marmet, S., Studer, J., Grazioli, V. S., & Gmel, G. (2018). Bidirectional associations between self-reported gaming disorder and Adult Attention Deficit Hyperactivity Disorder: Evidence from a sample of young Swiss men. *Frontiers in Psychiatry*, *9*, 649. doi:10.3389/fpsyt.2018.00649

Marmet, S., Studer, J., Wicki, M., Bertholet, N., Khazaal, Y., & Gmel, G. (2019). Unique versus shared associations between self-reported behavioral addictions and substance use disorders and mental health problems: A commonality analysis in a large sample of young Swiss men. *Journal of Behavioral Addictions*, *8*(4), 664-677. doi:10.1556/2006.8.2019.70

Maurya, K., & Dadhore, S. (2019). WAIT & WATCH THROUGH YOGA TECHNIQUES TO OVERCOME TENTATIVE INTERNET GAME DISORDER IN ADOLESCENT GROUPS. *International Journal of Adaptive Physical Education & Yoga*, *4*(5), 1-11.

Mentzoni, R. A., Brunborg, G. S., Molde, H., Myrseth, H., Skouverøe, K. J. M., Hetland, J., & Pallesen, S. (2011). Problematic video game use: estimated prevalence and associations with mental and physical health. *Cyberpsychology, Behavior, and Social Networking*, *14*(10), 591-596. doi:10.1089/cyber.2010.0260

Mills, D. J., Marchica, L., Keough, M. T., & Derevensky, J. L. (2020). Exploring differences in substance use among emerging adults at-risk for problem gambling, and/or problem video gaming. *International Gambling Studies*, *20*(3), 539-555. doi:10.1080/14459795.2020.1752768

Mills, D. J., Milyavskaya, M., Heath, N. L., & Derevensky, J. L. (2018). Gaming motivation and problematic video gaming: The role of needs frustration. *European Journal of Social Psychology*, *48*(4), 551-559. doi:10.1002/ejsp.2343

Molde, H., Holmøy, B., Merkesdal, A. G., Torsheim, T., Mentzoni, R. A., Hanns, D., Sagoe, D., & Pallesen, S. (2019). Are video games a gateway to gambling? A longitudinal study based on a representative Norwegian sample. *Journal of Gambling Studies*, *35*(2), 545-557. doi:10.1007/s10899-018-9781-z

Monacis, L., de Palo, V., Griffiths, M. D., & Sinatra, M. (2017). Exploring individual differences in online addictions: The role of identity and attachment. *International Journal of Mental Health and Addiction*, *15*(4), 853-868. doi:10.1007/s11469-017-9768-5

Monacis, L., De Palo, V., Griffiths, M. D., & Sinatra, M. (2017). Social networking addiction, attachment style, and validation of the Italian version of the Bergen Social Media Addiction Scale. *Journal of Behavioral Addictions*, *6*(2), 178-186. doi:10.1556/2006.6.2017.023

Monacis, L., Palo, V. d., Griffiths, M. D., & Sinatra, M. (2016). Validation of the internet gaming disorder scale–short-form (IGDS9-SF) in an Italian-speaking sample. *Journal of Behavioral Addictions*, *5*(4), 683-690. doi:10.1556/2006.5.2016.083

Morcos, M., Stavropoulos, V., Rennie, J. J., Clark, M., & Pontes, H. M. (2019). Internet gaming disorder: compensating as a Draenei in World of Warcraft. *International Journal of Mental Health and Addiction*, 1-17. doi:10.1007/s11469-019-00098-x

Moudiab, S., & Spada, M. M. (2019). The relative contribution of motives and maladaptive cognitions to levels of Internet Gaming Disorder. *Addictive Behaviors Reports*, *9*, 100160. doi:10.1016/j.abrep.2019.100160

Mueller, K. W., Dreier, M., Duven, E., Giralt, S., Beutel, M. E., & Woelfling, K. (2017). Adding Clinical Validity to the Statistical Power of Large-Scale Epidemiological Surveys on Internet Addiction in Adolescence: A Combined Approach to Investigate Psychopathology and Development-Specific Personality Traits Associated With Internet Addiction. *The Journal of Clinical Psychiatry*, *78*(3), 244-251. doi:10.4088/JCP.15m10447

Müller, K. W., Beutel, M., Egloff, B., & Wölfling, K. (2014). Investigating risk factors for Internet gaming disorder: a comparison of patients with addictive gaming, pathological gamblers and healthy controls regarding the big five personality traits. *European Addiction Research*, *20*(3), 129-136. doi:10.1159/000355832

Müller, K. W., Beutel, M. E., Dreier, M., & Wölfling, K. (2019). A clinical evaluation of the DSM-5 criteria for Internet Gaming Disorder and a pilot study on their applicability to further Internet-related disorders. *Journal of Behavioral Addictions*, *8*(1), 16-24. doi:10.1556/2006.7.2018.140

Müller, K. W., Glaesmer, H., Brähler, E., Woelfling, K., & Beutel, M. E. (2014). Prevalence of internet addiction in the general population: results from a German population-based survey. *Behaviour & Information Technology*, *33*(7), 757-766. doi:10.1080/0144929X.2013.810778

Müller, K. W., Janikian, M., Dreier, M., Wölfling, K., Beutel, M., Tzavara, C., Richardson, C., & Tsitsika, A. (2015). Regular gaming behavior and internet gaming disorder in European adolescents: results from a cross-national representative survey of prevalence, predictors, and psychopathological correlates. *European Child & Adolescent Psychiatry*, *24*(5), 565-574. doi:10.1007/s00787-014-0611-2

Myrseth, H., & Notelaers, G. (2018). A latent class approach for classifying the problem and disordered gamers in a group of adolescence. *Frontiers in Psychology*, *9*, 2273. doi:10.3389/fpsyg.2018.02273

Myrseth, H., Olsen, O. K., Borud, E. K., & Strand, L. Å. (2017). Predictors of gaming behavior among military peacekeepers–exploring the role of boredom and loneliness in relation to gaming problems. *Journal of Military Studies*, *8*(1), 1-10. doi:10.1515/jms-2017-0001

Myrseth, H., Olsen, O. K., Strand, L. Å., & Borud, E. K. (2017). Gaming behavior among conscripts: The role of lower psychosocial well-being factors in explaining gaming addiction. *Military Psychology*, *29*(2), 128-142. doi:10.1037/mil0000148

O’Farrell, D. L., Baynes, K.-L., Pontes, H. M., Griffiths, M. D., & Stavropoulos, V. (2020). Depression and Disordered Gaming: Does Culture Matter? *International Journal of Mental Health and Addiction*. doi:10.1007/s11469-020-00231-1

Onder, C., & Ardic, A. (2020). Problematic Internet Use and Digital Gaming in Adolescents: Problems, Nursing Interventions, and Outcomes. *Journal of Psychosocial Nursing and Mental Health Services, 58*(22), 51-59. doi:10.3928/02793695-20200918-02

Pan, Y.-C., Chiu, Y.-C., & Lin, Y.-H. (2019). Development of the problematic mobile gaming questionnaire and prevalence of mobile gaming addiction among adolescents in Taiwan. *Cyberpsychology, Behavior, and Social Networking*, *22*(10), 662-669. doi:10.1089/cyber.2019.0085

Peeters, M., Koning, I., Lemmens, J., & Eijnden, R. v. d. (2019). Normative, passionate, or problematic? Identification of adolescent gamer subtypes over time. *Journal of Behavioral Addictions*, *8*(3), 574-585. doi:10.1556/2006.8.2019.55

Peeters, M., Koning, I., & van den Eijnden, R. (2018). Predicting Internet Gaming Disorder symptoms in young adolescents: A one-year follow-up study. *Computers in Human Behavior*, *80*, 255-261. doi:10.1016/j.chb.2017.11.008

Phan, O., Prieur, C., Bonnaire, C., & Obradovic, I. (2020). Internet Gaming Disorder: Exploring Its Impact on Satisfaction in Life in PELLEAS Adolescent Sample. *International Journal of Environmental Research and Public Health*, *17*(1), 3. doi:10.3390/ijerph17010003

Pontes, H. M. (2017). Investigating the differential effects of social networking site addiction and Internet gaming disorder on psychological health. *Journal of Behavioral Addictions*, *6*(4), 601-610. doi:10.1556/2006.6.2017.075

Pontes, H. M., & Griffiths, M. D. (2015). Measuring DSM-5 Internet gaming disorder: Development and validation of a short psychometric scale. *Computers in Human Behavior*, *45*, 137-143. doi:10.1016/j.chb.2014.12.006

Pontes, H. M., & Griffiths, M. D. (2016). Portuguese validation of the internet gaming disorder scale–short-form. *Cyberpsychology, Behavior, and Social Networking*, *19*(4), 288-293. doi:10.1089/cyber.2015.0605

Pontes, H. M., Macur, M., & Griffiths, M. D. (2016). Internet Gaming Disorder among Slovenian primary schoolchildren: Findings from a nationally representative sample of adolescents. *Journal of Behavioral Addictions*, *5*(2), 304-310. doi:10.1556/2006.5.2016.042

Pontes, H. M., Schivinski, B., Brzozowska-Woś, M., & Stavropoulos, V. (2019). Laxer clinical criteria for gaming disorder may hinder future efforts to devise an efficient diagnostic approach: A tree-based model study. *Journal of Clinical Medicine*, *8*(10), 1730. doi:10.3390/jcm8101730

Pontes, H. M., Stavropoulos, V., & Griffiths, M. D. (2017). Measurement invariance of the internet gaming disorder scale–short-form (IGDS9-SF) between the United States of America, India and the United Kingdom. *Psychiatry Research*, *257*, 472-478. doi:10.1016/j.psychres.2017.08.013

Qin, L., Cheng, L., Hu, M., Liu, Q., Tong, J., Hao, W., Luo, T., & Liao, Y. (2020). Clarification of the cut-off score for nine-item Internet Gaming Disorder Scale–Short Form (IGDS9-SF) in a Chinese context. *Frontiers in Psychiatry*, *11*, 470*.* doi:10.3389/fpsyt.2020.00470

Rao, J., Wang, H., Pang, M., Yang, J., Zhang, J., Ye, Y., Chen, X., Wang, S., & Dong, X. (2019). Cyberbullying perpetration and victimisation among junior and senior high school students in Guangzhou, China. *Injury Prevention*, *25*(1), 13-19. doi:10.1136/injuryprev-2016-042210

Reer, F., Festl, R., & Quandt, T. (2020). Investigating problematic social media and game use in a nationally representative sample of adolescents and younger adults. *Behaviour & Information Technology*, 1-14. doi:10.1080/0144929X.2020.1724333

Sahin, M., Gumus, Y. Y., & Dincel, S. (2016). Game addiction and academic achievement. *Educational Psychology*, *36*(9), 1533-1543. doi:10.1080/01443410.2014.972342

Saiful Islam, M., Rahman, M. E., Moonajilin, M. S., & Griffiths, M. D. (2020). Validation and evaluation of the psychometric properties of bangla nine-item internet disorder scale–short form. *Journal of Addictive Diseases*, *38*(4), 540-549. doi:10.1080/10550887.2020.1799134

Salam, Z., Sadiq, Z., Tajamul, U., Sethi, M. R., & Irfan, M. (2019). Internet gaming disorder in students of peshawar: A cross sectional survey. *Journal of Ayub Medical College Abbottabad*, *31*(4), 548-552.

Sánchez-Iglesias, I., Bernaldo-de-Quirós, M., Labrador, F. J., Puig, F. J. E., Labrador, M., & Fernández-Arias, I. (2020). Spanish Validation and Scoring of the Internet Gaming Disorder Scale-Short-Form (IGDS9-SF). *The Spanish Journal of Psychology, 23*, E22. doi:10.1017/SJP.2020.26

Sariyska, R., Lachmann, B., Markett, S., Reuter, M., & Montag, C. (2017). Individual differences in implicit learning abilities and impulsive behavior in the context of Internet addiction and Internet Gaming Disorder under the consideration of gender. *Addictive Behaviors Reports*, *5*, 19-28. doi:10.1016/j.abrep.2017.02.002

Savci, M., & Aysan, F. (2017). Technological addictions and social connectedness: predictor effect of internet addiction, social media addiction, digital game addiction and smartphone addiction on social connectedness. *Dusunen Adam: Journal of Psychiatry & Neurological Sciences*, *30*(3). 202-216. doi:10.5350/DAJPN2017300304

Severo, R. B., Barbosa, A. P. P. N., Fouchy, D. R. C., da Cunha Coelho, F. M., Pinheiro, R. T., de Figueiredo, V. L. M., de Siqueira Afonso, V., Pontes, H. M., & Pinheiro, K. A. T. (2020). Development and psychometric validation of internet gaming disorder scale-short-form (IGDS9-SF) in a Brazilian sample. *Addictive behaviors*, *103*, 106191. doi: 10.1016/j.addbeh.2019.106191.

Sharma, M., Anand, N., Marimuthu, P., Suma, N., Murthy, K., Thakur, P., Singh, P., Ajith, S., John, N., Mondal, I., Biswas, A., Archana, R., Vishwakarma, A., Tadpatrikar, A., & Ahuja, S. (2020). Anxiety as a predictor of gaming disorder among young adults. *Indian Journal of Social Psychiatry*, *36*(3), 254-257. doi:10.4103/ijsp.ijsp_129_19

Shibuya, A., Teramoto, M., Shoun, A., & Akiyama, K. (2019). Long-term effects of in-game purchases and event game mechanics on young mobile social game players in Japan. *Simulation & Gaming*, *50*(1), 76-92. doi:10.1177/1046878118819677

Sigerson, L., Li, A. Y.-L., Cheung, M. W.-L., & Cheng, C. (2017). Examining common information technology addictions and their relationships with non-technology-related addictions. *Computers in Human Behavior*, *75*, 520-526. doi:10.1016/j.chb.2017.05.041

Singh, S., Dahiya, N., Singh, A. B., Kumar, R., & Balhara, Y. P. S. (2019). Gaming disorder among medical college students from India: Exploring the pattern and correlates. *Industrial psychiatry journal*, *28*(1), 107-114. doi:10.4103/ipj.ipj_96_18

Sioni, S. R., Burleson, M. H., & Bekerian, D. A. (2017). Internet gaming disorder: Social phobia and identifying with your virtual self. *Computers in Human Behavior*, *71*, 11-15. doi:10.1016/j.chb.2017.01.044.

Snodgrass, J. G., Dengah, H. F., Polzer, E., & Else, R. (2019). Intensive online videogame involvement: A new global idiom of wellness and distress. *Transcultural Psychiatry*, *56*(4), 748-774. doi:10.1177/1363461519844356

Snodgrass, J. G., Dengah II, H. F., Lacy, M. G., Else, R. J., Polzer, E. R., Arevalo, J. M., & Cole, S. W. (2018). Social genomics of healthy and disordered internet gaming. *American Journal of Human Biology*, *30*(5), e23146. doi:10.1002/ajhb.23146

Soraci, P., Ferrari, A., Bonanno, E., Repice, E., & Griffiths, M. D. (2020). Psychometric Validation of the Internet Disorder Scale–Short Form in an Italian Adult Sample. *International Journal of Mental Health and Addiction*. doi:10.1007/s11469-020-00279-z

Šporčić, B., & Glavak-Tkalić, R. (2018). The relationship between online gaming motivation, self-concept clarity and tendency toward problematic gaming. *Cyberpsychology: Journal of Psychosocial Research on Cyberspace*, *12*(1). doi:10.5817/CP2018-1-4

Stavropoulos, V., Adams, B. L., Beard, C. L., Dumble, E., Trawley, S., Gomez, R., & Pontes, H. M. (2019). Associations between attention deficit hyperactivity and internet gaming disorder symptoms: Is there consistency across types of symptoms, gender and countries? *Addictive Behaviors Reports*, *9*, 100158. doi:10.1016/j.abrep.2018.100158

Stavropoulos, V., Anderson, E. E., Beard, C., Latifi, M. Q., Kuss, D., & Griffiths, M. (2019). A preliminary cross-cultural study of Hikikomori and Internet Gaming Disorder: The moderating effects of game-playing time and living with parents. *Addictive Behaviors Reports*, *9*, 100137. doi:10.1016/j.abrep.2018.10.001

Stavropoulos, V., Bamford, L., Beard, C., Gomez, R., & Griffiths, M. D. (2019). Test-retest measurement invariance of the nine-item internet gaming disorder scale in two countries: a preliminary longitudinal study. *International Journal of Mental Health and Addiction*. doi:10.1007/s11469-019-00099-w

Stavropoulos, V., Beard, C., Griffiths, M. D., Buleigh, T., Gomez, R., & Pontes, H. M. (2018). Measurement invariance of the internet gaming disorder scale–short-form (IGDS9-SF) between Australia, the USA, and the UK. *International Journal of Mental Health and Addiction*, *16*(2), 377-392. doi:10.1007/s11469-017-9786-3

Stavropoulos, V., Burleigh, T. L., Beard, C. L., Gomez, R., & Griffiths, M. D. (2019). Being there: a preliminary study examining the role of presence in internet gaming disorder. *International Journal of Mental Health and Addiction*, *17*(4), 880-890. doi:10.1007/s11469-018-9891-y

Stavropoulos, V., Dumble, E., Cokorilo, S., Griffiths, M. D., & Pontes, H. M. (2019). The physical, emotional, and identity user-avatar association with disordered gaming: A pilot study. *International Journal of Mental Health and Addiction*. doi:10.1007/s11469-019-00136-8

Stavropoulos, V., Pontes, H. M., Gomez, R., Schivinski, B., & Griffiths, M. (2020). Proteus Effect Profiles: how Do they Relate with Disordered Gaming Behaviours? *Psychiatric Quarterly*, *91*(3), 615-628. doi:10.1007/s11126-020-09727-4

T’ng, S. T., & Pau, K. (2020). Identification of avatar mediates the associations between motivations of gaming and internet gaming disorder among the Malaysian youth. *International Journal of Mental Health and Addiction*. doi:10.1007/s11469-020-00229-9

T’ng, S. T., & Pau, K. (2020). Validation of a translated Internet Gaming Disorder Scale (Short Form) and measurement invariance across sex groups in Malaysian samples. *Current Psychology*, *39*, 1977-1989. doi:10.1007/s12144-020-00668-6

T’ng, S. T., Ho, K. H., Sim, D. E., Yu, C. H., & Wong, P. Y. (2020). The mediating effect of Internet gaming disorder's symptoms on loneliness and aggression among undergraduate students and working adults in Malaysia. *PsyCh Journal*, *9*(1), 96-107. doi:10.1002/pchj.320

Teng, Z., Pontes, H. M., Nie, Q., Xiang, G., Griffiths, M. D., & Guo, C. (2020). Internet gaming disorder and psychosocial well-being: A longitudinal study of older-aged adolescents and emerging adults. *Addictive Behaviors*, *110*, 106530. doi:10.1016/j.addbeh.2020.106530

Throuvala, M. A., Janikian, M., Griffiths, M. D., Rennoldson, M., & Kuss, D. J. (2019). The role of family and personality traits in Internet gaming disorder: A mediation model combining cognitive and attachment perspectives. *Journal of Behavioral Addictions*, *8*(1), 48-62. doi:10.1556/2006.8.2019.05

Tian, Y., Zhang, S., Wu, R., Wang, P., Gao, F., & Chen, Y. (2018). Association between specific internet activities and life satisfaction: the mediating effects of loneliness and depression. *Frontiers in Psychology*, *9*, 1181. doi:10.3389/fpsyg.2018.01181

Uçar, H. N., Çetin, F. H., Ersoy, S. A., Güler, H. A., Kılınç, K., & Türkoğlu, S. (2020). Risky cyber behaviors in adolescents with depression: A case control study. *Journal of Affective Disorders*, *270*, 51-58. doi:10.1016/j.jad.2020.03.046

Vally, Z. (2020). Symptoms of Internet Gaming Disorder, Inattention, and Impulsivity: a Cross-Sectional Study Conducted in the United Arab Emirates. *Psychiatric Quarterly*, *92*, 301-310. doi:10.1007/s11126-020-09799-2

Van Den Eijnden, R., Koning, I., Doornwaard, S., Van Gurp, F., & Ter Bogt, T. (2018). The impact of heavy and disordered use of games and social media on adolescents’ psychological, social, and school functioning. *Journal of Behavioral Addictions*, *7*(3), 697-706. doi:10.1556/2006.7.2018.65

van Rooij, A. J., Schoenmakers, T. M., Van den Eijnden, R. J., Vermulst, A. A., & van de Mheen, D. (2012). Video game addiction test: validity and psychometric characteristics. *Cyberpsychology, Behavior, and Social Networking*, *15*(9), 507-511. doi:10.1089/cyber.2012.0007

Vanzoelen, D., & Caltabiano, M. L. (2016). The role of social anxiety, the behavioural inhibition system and depression in online gaming addiction in adults. *Journal of Gaming & Virtual Worlds*, *8*(3), 231-245. doi:10.1386/jgvw.8.3.231_1

Wang, C.-W., Chan, C. L., Mak, K.-K., Ho, S.-Y., Wong, P. W., & Ho, R. T. (2014). Prevalence and correlates of video and Internet gaming addiction among Hong Kong adolescents: A pilot study. *The Scientific World Journal*, *2014*. doi:10.1155/2014/874648

Wang, H.-Y., & Cheng, C. (2021). New perspectives on the prevalence and associated factors of gaming disorder in Hong Kong community adults: A generational approach. *Computers in Human Behavior*, *114*. doi:10.1016/j.chb.2020.106574

Wang, H.-Y., Sigerson, L., & Cheng, C. (2019). Digital nativity and information technology addiction: Age cohort versus individual difference approaches. *Computers in Human Behavior*, *90*, 1-9. doi:10.1016/j.chb.2018.08.031

Wang, P., Yan, Y., Gao, F., Zhang, R., Wang, J., Zhan, X., & Tian, Y. (2020). The Effect of Shyness on Adolescent Network Problem Behavior: The Role of Gender and Loneliness. *Frontiers in Psychology*, *11*, 803. doi:10.3389/fpsyg.2020.00803

Wang, Z., Xie, Q., Xin, M., Wei, C., Yu, C., Zhen, S., Liu, S., Wang, J., & Zhang, W. (2020). Cybervictimization, Depression, and Adolescent Internet Addiction: The Moderating Effect of Prosocial Peer Affiliation. *Frontiers in Psychology*, *11*, 2527. doi: 10.3389/fpsyg.2020.572486

Wartberg, L., Kriston, L., & Thomasius, R. (2017). The prevalence and psychosocial correlates of internet gaming disorder: analysis in a nationally representative sample of 12-to 25-Year-Olds. *Deutsches Ärzteblatt International*, *114*(25), 419-424. doi: 10.3238/arztebl.2017.0419

Wartberg, L., Kriston, L., & Thomasius, R. (2020). Internet gaming disorder and problematic social media use in a representative sample of German adolescents: Prevalence estimates, comorbid depressive symptoms and related psychosocial aspects. *Computers in Human Behavior*, *103*, 31-36. doi:10.1016/j.chb.2019.09.014.

Wartberg, L., Kriston, L., Zieglmeier, M., Lincoln, T., & Kammerl, R. (2019). A longitudinal study on psychosocial causes and consequences of Internet gaming disorder in adolescence. *Psychological Medicine*, *49*(2), 287-294. doi:10.1017/S003329171800082X

Wartberg, L., Zieglmeier, M., & Kammerl, R. (2019). Accordance of adolescent and parental ratings of Internet gaming disorder and their associations with psychosocial aspects. *Cyberpsychology, Behavior, and Social Networking*, *22*(4), 264-270. doi: 10.1089/cyber.2018.0456

Wittek, C. T., Finserås, T. R., Pallesen, S., Mentzoni, R. A., Hanss, D., Griffiths, M. D., & Molde, H. (2016). Prevalence and predictors of video game addiction: A study based on a national representative sample of gamers. *International Journal of Mental Health and Addiction*, *14*(5), 672-686. doi:10.1007/s11469-015-9592-8

Wölfling, K., Müller, K. W., Dreier, M., Ruckes, C., Deuster, O., Batra, A., Mann, K., Musalek, M., Schuster, A., & Lemenager, T. (2019). Efficacy of short-term treatment of internet and computer game addiction: A randomized clinical trial. *JAMA Psychiatry*, *76*(10), 1018-1025. doi:10.1001/jamapsychiatry.2019.1676

Wong, H. Y., Mo, H. Y., Potenza, M. N., Chan, M. N. M., Lau, W. M., Chui, T. K., Pakpour, A. H., & Lin, C.-Y. (2020). Relationships between severity of internet gaming disorder, severity of problematic social media use, sleep quality and psychological distress. *International Journal of Environmental Research and Public Health*, *17*(6), 1879. doi:10.3390/ijerph17061879

Wu, T.-Y., Lin, C.-Y., Årestedt, K., Griffiths, M. D., Broström, A., & Pakpour, A. H. (2017). Psychometric validation of the Persian nine-item Internet Gaming Disorder Scale–Short Form: Does gender and hours spent online gaming affect the interpretations of item descriptions? *Journal of Behavioral Addictions*, *6*(2), 256-263. doi:10.1556/2006.6.2017.025

Yam, C.-W., Pakpour, A. H., Griffiths, M. D., Yau, W.-Y., Lo, C.-L. M., Ng, J. M., Lin, C.-Y., & Leung, H. (2019). Psychometric testing of three Chinese online-related addictive behavior instruments among Hong Kong university students. *Psychiatric Quarterly*, *90*(1), 117-128. doi:10.1007/s11126-018-9610-7

Zhou, X., Wu, R., Liu, C., Kou, J., Chen, Y., Pontes, H. M., Yao, D., Kendrick, K. M., Becker, B., & Montag, C. (2020). Higher levels of (Internet) Gaming Disorder symptoms according to the WHO and APA frameworks associate with lower striatal volume. *Journal of Behavioral Addictions*, *9*(3), 598–605. doi:10.1556/2006.2020.00066
