# Supplementary material for: Reliability, and Convergent and Discriminant Validity of Gaming Disorder Scales: A Meta-Analysis
Source: Front Psychol. 2021 Dec 7;12:764209. doi: 10.3389/fpsyg.2021.764209 (PMC8689178; doi:10.3389/fpsyg.2021.764209)
Supplement: Supplementary file 5 [file Data_Sheet_1.docx]

**Supplementary material**

*Supplementary Table 1*. Categorical moderator analysis for reliability generalization

| Moderator | *QM* | *df* | *p* | Subgroup | *k* | $\alpha_{trf}$ | *SE* | 95% CI | $I^{2}$(%) |
| --- | --- | --- | --- | --- | --- | --- | --- | --- | --- |
| Measure | 51.56 | 4 | **< .01***** |  |  |  |  |  |  |
|  |  |  |  | IGDS9-SF | 90 | 2.205 | 0.047 | [2.109, 2.289] | 98.46 |
|  |  |  |  | GAS-7 | 58 | 1.814 | 0.058 | [1.701, 1.927] | 98.93 |
|  |  |  |  | Lemmens IGD-9 | 20 | 1.637 | 0.098 | [1.446, 1.828] | 98.70 |
|  |  |  |  | IGDT-10 | 16 | 1.696 | 0.110 | [1.442, 1.882] | 99.66 |
|  |  |  |  | AICA | 9 | 1.919 | 0.146 | [1.634, 2.203] | 99.72 |

*Note. QM =* test of moderator; *k* = number of reported effect sizes; $\alpha_{trf}$ = transformed mean Cronbach’s alpha coefficient;

$SE$ = standard deviation for $\alpha_{trf}$; $I^{2}$= total heterogeneity / total variability; *p* < .001. ***

*Supplementary Table 2*. Categorical moderator analysis for validity generalization

| Variable | Moderator | Included subgroups (*k* ≥ 4) | *QM* | *df* | *p* | Subgroup | *k* | *r* | *SE* | 95% CI | $I^{2}$(%) |
| --- | --- | --- | --- | --- | --- | --- | --- | --- | --- | --- | --- |
| Depression | Measure | GAS-7, IGDS9-SF, IGDT10 | 5.17 | 2 | .75 |  |  |  |  |  |  |
|  | Sample | Adolescents, Adults, Both, | 1.10 | 2 | .58 |  |  |  |  |  |  |
|  | Location | Asia, Europe | 1.13 | 1 | .29 |  |  |  |  |  |  |
| Anxiety | Measure | GAS-7, IGDS9-SF | 4.95 | 1 | **.02 *** | GAS-7 | 11 | .233 | 0.028 | [.178, .289] | 92 |
|  |  |  |  |  |  | IGDS9-SF | 19 | .326 | 0.030 | [.266, .385] | 94 |
|  | Sample | Adolescents, Adults, Both, | 4.04 | 2 | .13 |  |  |  |  |  |  |
|  | Location | Asia, Europe | 1.82 | 1 | .18 |  |  |  |  |  |  |
| Aggression | Sample | Adults, Both, Adolescents | 3.28 | 2 | .19 |  |  |  |  |  |  |
|  | Location | Asia, Europe | 8.01 | 1 | **< .01 **** | Asia | 7 | .379 | 0.025 | [.330, .428] | 78 |
|  |  |  |  |  |  | Europe | 11 | .243 | 0.041 | [.163, .324] | 97 |
| QoL | Measure | GAS-7, IGDS9-SF | 0.10 | 1 | .75 |  |  |  |  |  |  |
|  | Sample | Adolescents, Adults, Both, | 1.51 | 1 | .22 |  |  |  |  |  |  |
|  | Location | Asia, Europe | 0.53 | 1 | .46 |  |  |  |  |  |  |
| Loneliness | Measure | GAS-7, IGDS9-SF | 0.52 | 1 | .47 |  |  |  |  |  |  |
|  | Sample | Adolescents, Adults, Both, | 1.44 | 2 | .49 |  |  |  |  |  |  |
|  | Location | Asia, Europe | 2.72 | 1 | .10 † | Asia | 10 | .334 | 0.063 | [.211, .457] | 92 |
|  |  |  |  |  |  | Europe | 7 | .221 | 0.027 | [.169, .273] | 64 |
| Internet addiction | Measure | GAS-7, IGDS9-SF | 3.84 | 1 | .05 † | GAS-7 | 9 | .552 | 0.077 | [.400, .703] | 97 |
|  |  |  |  |  |  | IGDS9-SF | 8 | .782 | 0.089 | [.608, .955] | 98 |
|  | Sample | Adolescents, Adults, Both, | 2.66 | 2 | .26 |  |  |  |  |  |  |
|  | Location | Asia, Europe | 0.06 | 1 | .80 |  |  |  |  |  |  |
| Game playtime | Measure | GAS-7, IGDS9-SF, IGDT10,  Lemmens IGD-9 | 7.06 | 3 | .07 † | GAS-7 | 17 | .480 | 0.040 | [.405, .554] | 96 |
|  |  |  |  |  |  | IGDS9-SF | 23 | .431 | 0.044 | [.345, .517] | 95 |
|  |  |  |  |  |  | IGDT-10 | 5 | .217 | 0.092 | [.036, .397] | 99 |
|  |  |  |  |  |  | Lemmens IGD-9 | 8 | .416 | 0.119 | [.183, .650] | 98 |
|  | Sample | Adolescents, Adults, Both, | 5.56 | 2 | .06 † | Adolescents | 14 | .504 | 0.041 | [.423, .584] | 96 |
|  |  |  |  |  |  | Adults | 14 | .464 | 0.079 | [.310, .619] | 97 |
|  |  |  |  |  |  | Both | 24 | .373 | 0.038 | [.298, .448] | 95 |
|  | Location | Asia, Europe, North America | 0.90 | 2 | .63 |  |  |  |  |  |  |

*Note. QM =* test of moderator*; k* = number of reported effect sizes; $r$ = estimated mean effect sizes (correlation coefficient); $SE$ = standard deviation for $r_{obs}$;

$I^{2}$= total heterogeneity / total variability; estimates of each subgroup were presented when omnibus test of moderator *QM* indicates the subgroup differences (*p* < .05);

unavailable subgroup analyses due to insufficient number of studies were omitted from the table; *p* <.01. **, *p* <.05. *. *p* <.10. †

*Supplementary Table 3*. Continuous moderator analysis for validity generalization

| Variable | *k* | *QM* | *df* | *p* | $\tau^{2}$ | $I^{2}$(%) | Estimate | *b* | *SE* | 95% CI |
| --- | --- | --- | --- | --- | --- | --- | --- | --- | --- | --- |
| Depression | 45 | 1.73 | 1 | .19 | 0.018 | 96 | Intercept | 0.2604 | 0.0659 | [0.1331, 0.3877] |
|  |  |  |  |  |  |  | Male (%) | 0.0014 | 0.0010 | [-0.0007, 0.0034] |
| Anxiety | 37 | 0.00 | 1 | .98 | 0.017 | 97 | Intercept | 0.3006 | 0.0715 | [0.1605, 0.4408] |
|  |  |  |  |  |  |  | Male (%) | 0.0000 | 0.0011 | [-0.0022, 0.0023] |
| Aggression | 19 | 3.35 | 1 | .07 † | 0.013 | 96 | Intercept | 0.4547 | 0.0949 | [0.2687, 0.6407] |
|  |  |  |  |  |  |  | Male (%) | -0.0028 | 0.015 | [-0.0057, 0.0002] |
| QoL | 18 | 0.60 | 1 | .43 | 0.044 | 98 | Intercept | -0.0879 | 0.1788 | [-0.4383, 0.2625] |
|  |  |  |  |  |  |  | Male (%) | -0.0022 | 0.0029 | [-0.0078, 0.0034] |
| Loneliness | 18 | 1.60 | 1 | .21 | 0.0257 | 97 | Intercept | 0.1282 | 0.1404 | [-0.1471, 0.4034] |
|  |  |  |  |  |  |  | Male (%) | 0.0027 | 0.0021 | [-0.0015, 0.0069] |
| Internet addiction | 20 | 1.75 | 1 | .19 | 0.060 | 98 | Intercept | 0.8424 | 0.1665 | [0.5160, 1.1688] |
|  |  |  |  |  |  |  | Male (%) | -0.0034 | 0.0026 | [-0.0084, 0.0016] |
| Game playtime | 53 | 4.11 | 1 | **.04 *** | 0.045 | 98 | Intercept | 0.6302 | 0.1061 | [0.4223, 0.8381] |
|  |  |  |  |  |  |  | Male (%) | -0.0033 | 0.0016 | [-0.0065, -0.0001] |

*Note. k* = number of reported effect sizes; *QM =* test of moderator; $\tau^{2}$= estimated amount of total heterogeneity; $I^{2}$= total heterogeneity / total variability;

$b$ = estimated beta; $SE$ = standard deviation for $b$; for every one-unit (one percent point) increase in the predictor variable (% of male participants),

the outcome variable increase by the beta coefficient value; *p* <.05. *. *p* <.10. †

*Supplementary Figure 1.* Forest plots of reliability generalization


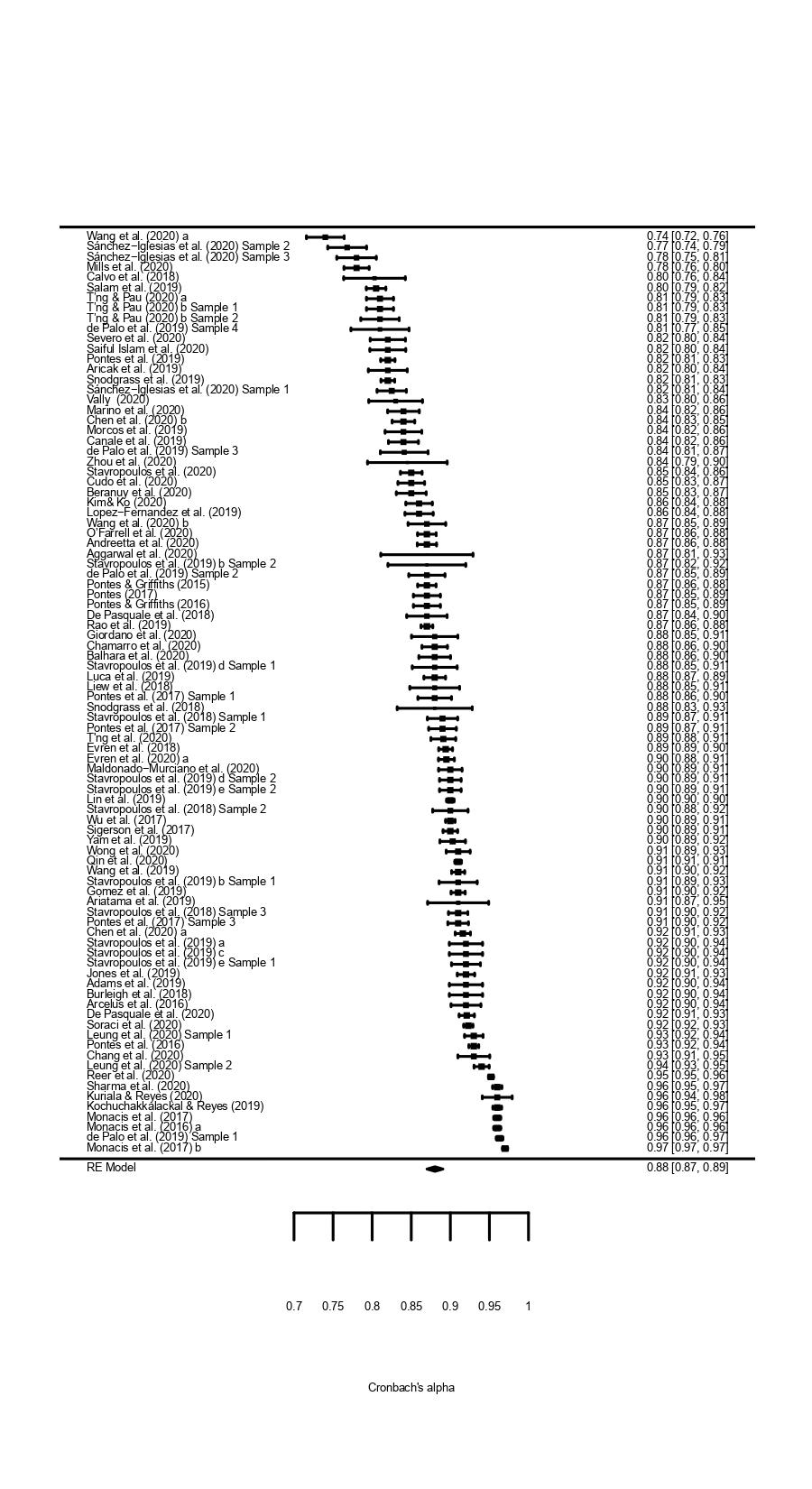


(a) IGDS9-SF


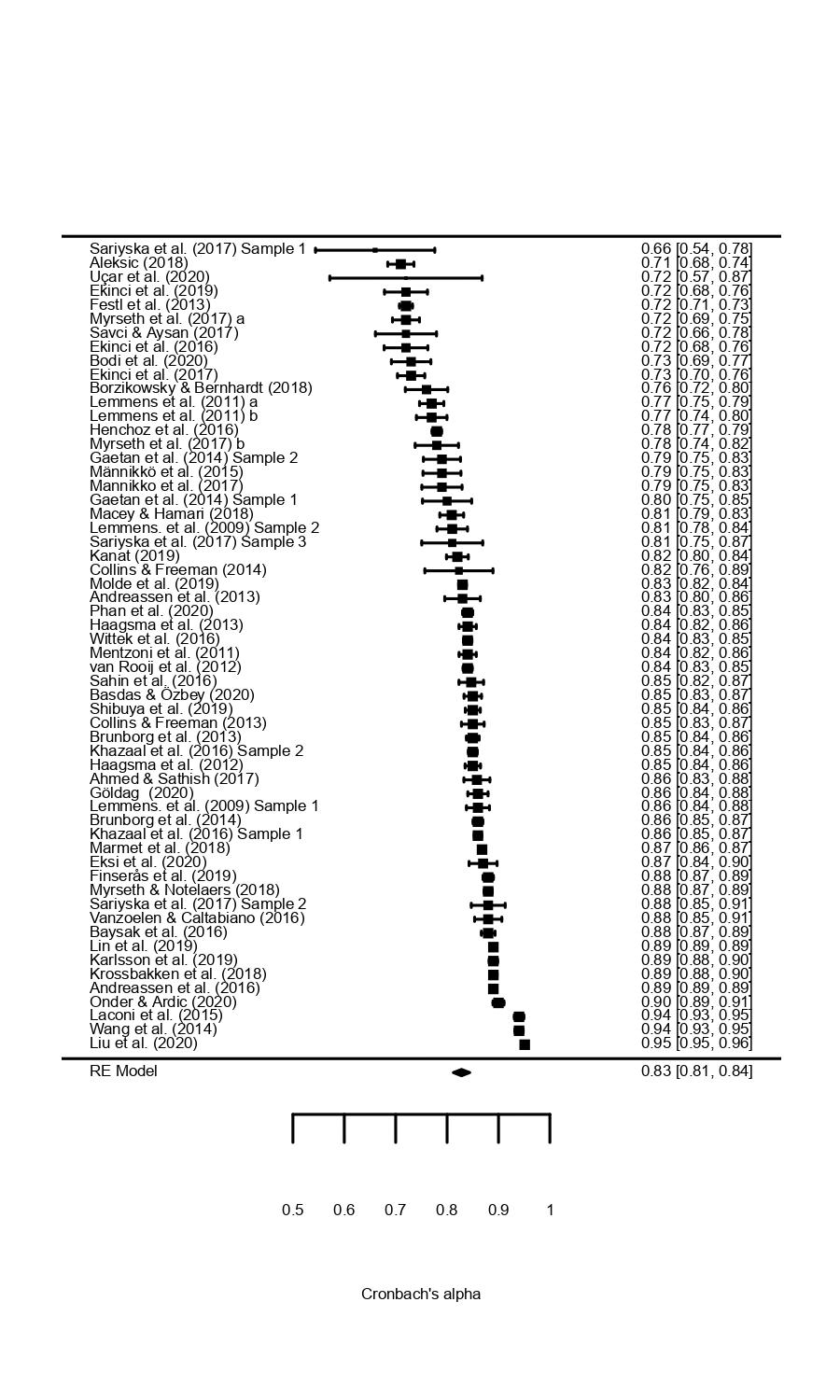

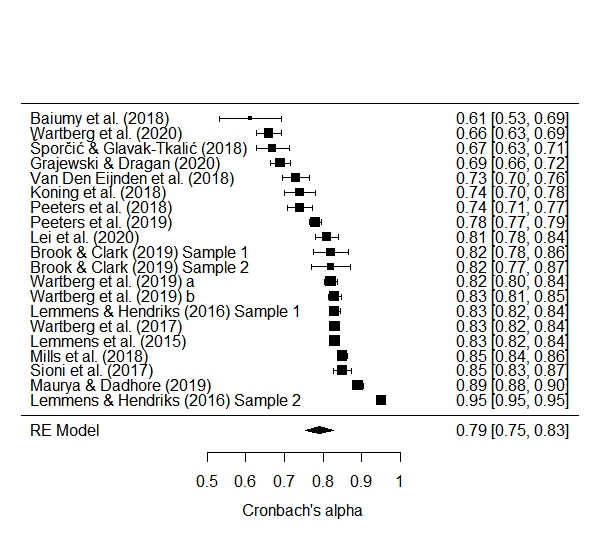


(b) GAS-7


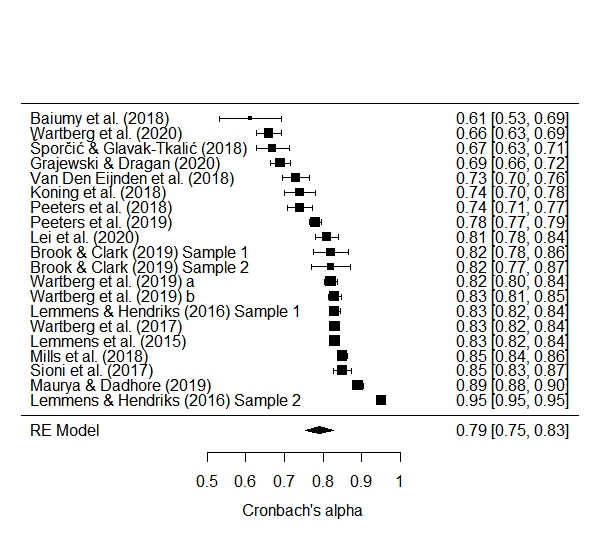
(c) Lemmens IGD-9


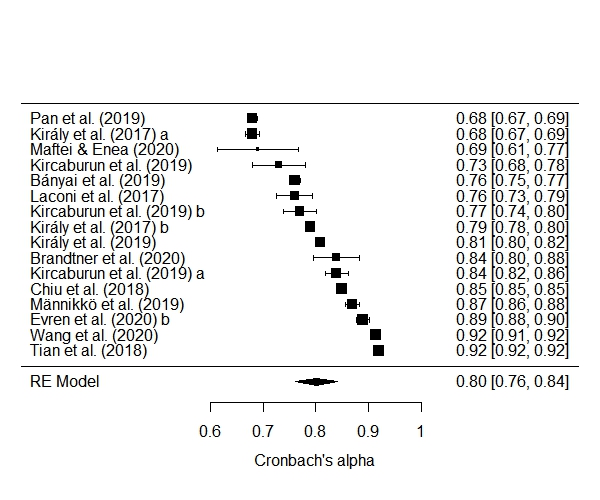


(d) IGDT-10


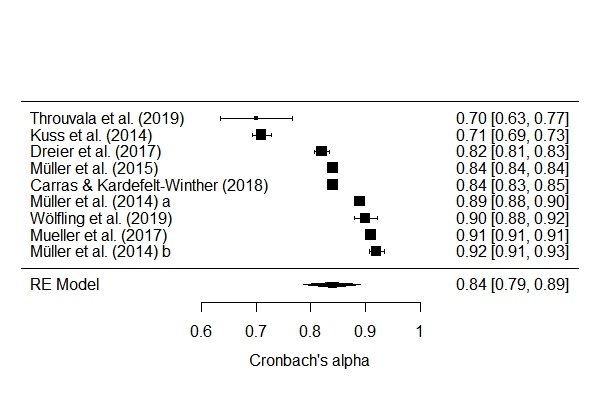


(e) AICA

*Supplementary Figure 2*. Funnel plots of reliability generalization


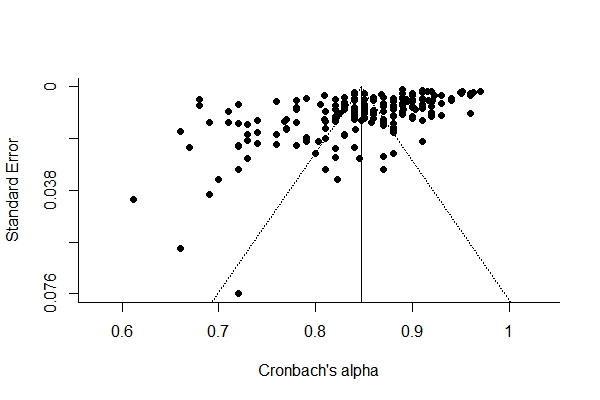


(a) Overall reliability generalization


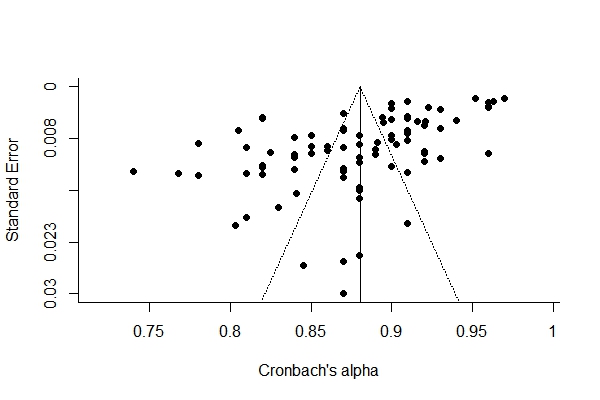


(b) IGDS9-SF


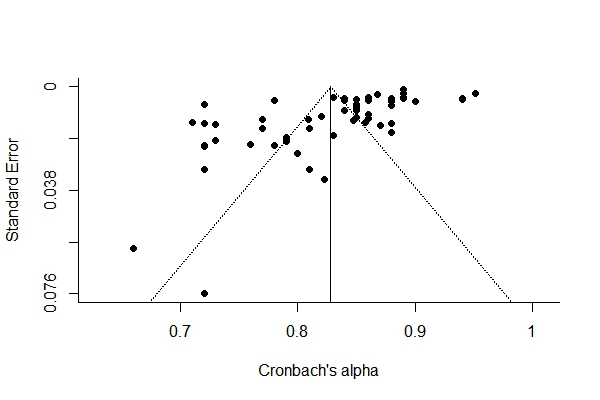


(c) GAS-7


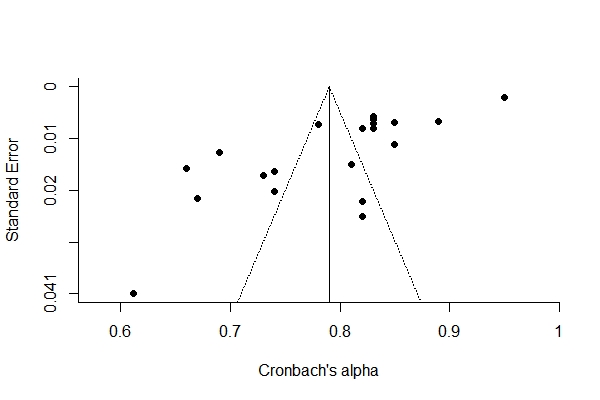


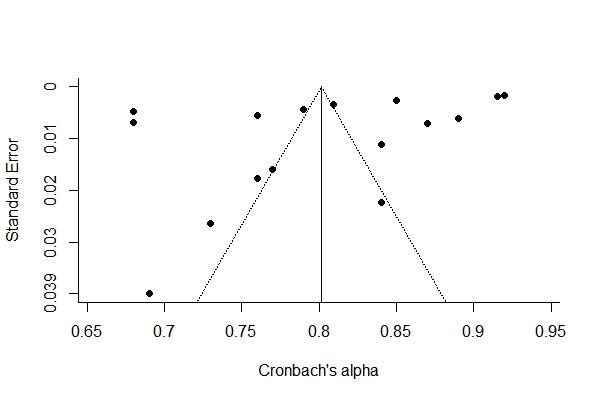
(d) Lemmens IGD-9

(e) IGDT-10


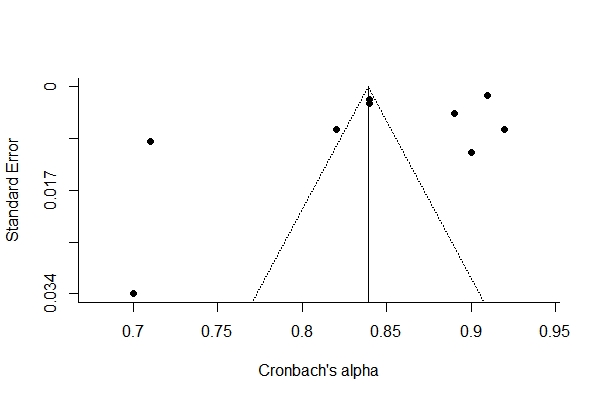


(f) AICA

*Supplementary Figure 3*. Funnel plots of validity generalization.


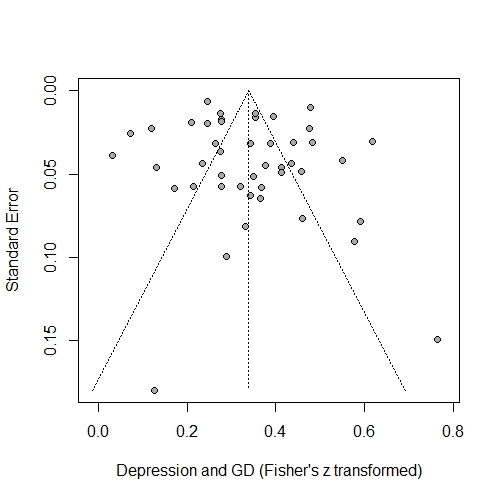


1. Depression and GD


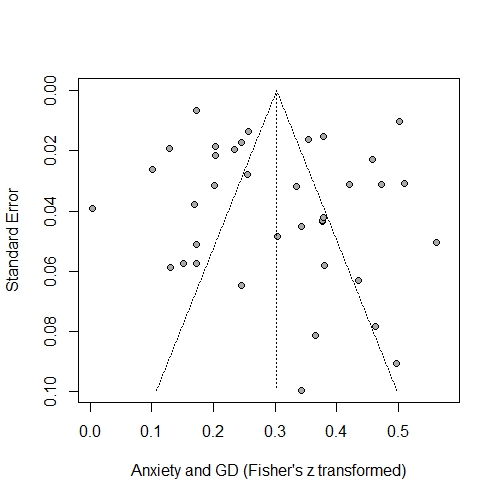


1. Anxiety and GD


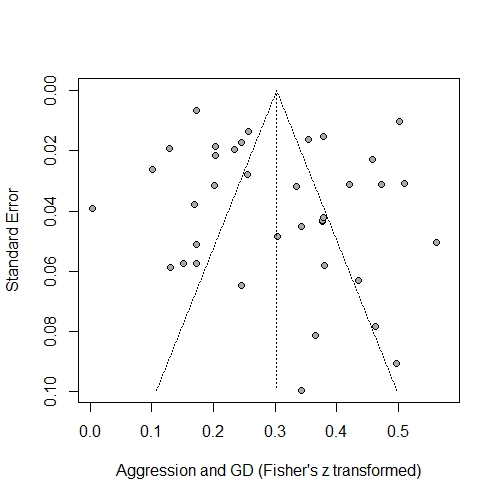


1. Aggression and GD


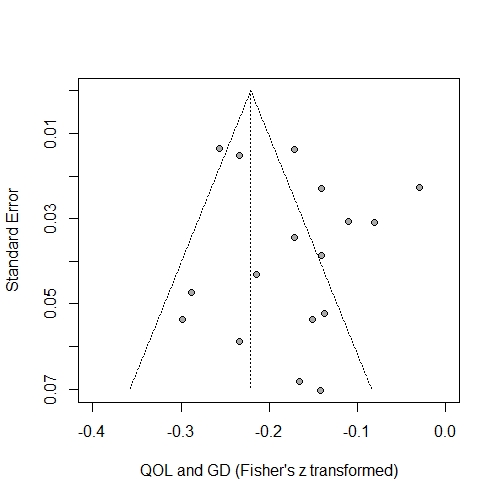


1. Quality of life and GD


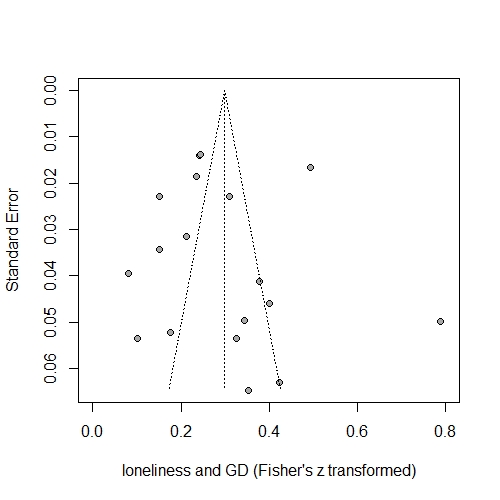


1. Loneliness and GD


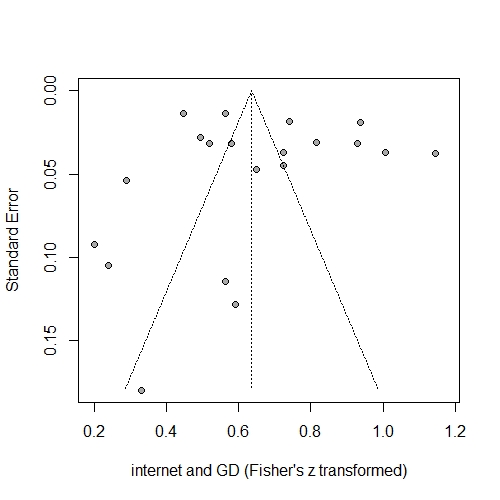


1. Internet addiction and GD


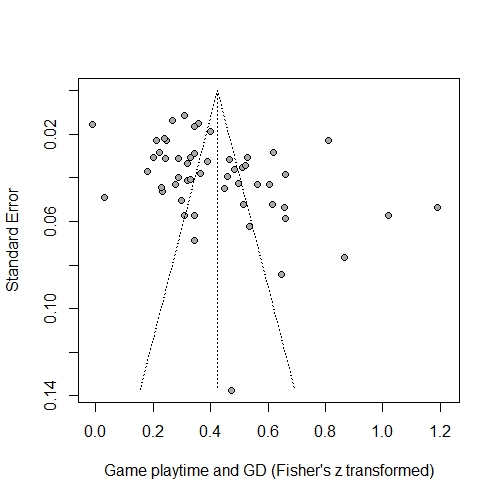


1. Game playtime and GD

*Supplementary Figure 4*. Cumulative meta-analysis for game playtime and GD

**
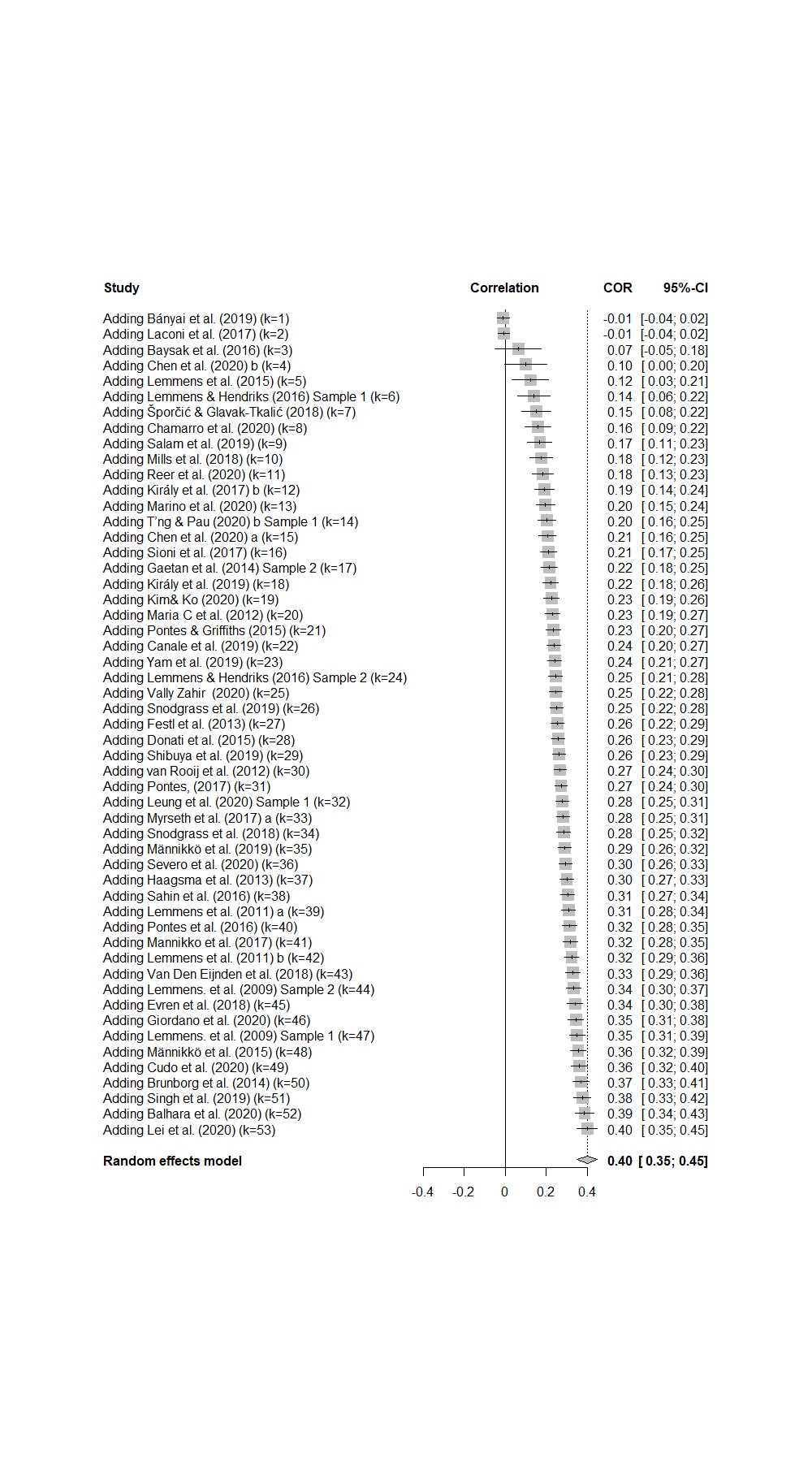
**
